# Supplementary material for: The psychological, computational, and neural foundations of indebtedness
Source: Nat Commun. 2024 Jan 2;15:68. doi: 10.1038/s41467-023-44286-9 (PMC10762097; doi:10.1038/s41467-023-44286-9)
Supplement: Supplementary file 1 — Supplementary Information [file 41467_2023_44286_MOESM1_ESM.pdf]

Supplementary Information for

# The psychological, computational, and neural foundations of indebtedness

*Xiaoxue Gao*, Eshin Jolly, Hongbo Yu, Huiying Liu, Xiaolin Zhou*, Luke J. Chang**

*Correspondence to:

Xiaoxue Gao (gxx114455@gmail.com or xxgao@psy.ecnu.edu.cn),

Xiaolin Zhou (xz104@psy.ecnu.edu.cn),

and Luke J. Chang (luke.j.chang@dartmouth.edu)

**This PDF file includes:**

**Supplementary Methods** ........ 2

Experimental procedures ........ 2

Data analyses ........ 8

Online questionnaire for Study 1 ........ 24

**Supplementary Notes** ........ 30

Predicting behaviors using Communal and Obligation Factors ........ 30

Results of efficiency manipulation ........ 30

**Supplementary References** ........ 32

**Supplementary Figures** ........ 37

**Supplementary Tables** ........ 45

## Supplementary Methods

### Experimental procedures

***Study 1 - Online Questionnaire***

**Online questionnaire.** Events were required to be clearly recalled and to have occurred within the past year. Appraisal questions included: "To what extent do you think the benefactor expected you to repay? (i.e., second-order belief; Q. 15)”, and "To what extent do you think the benefactor cared about your welfare when helping you? (i.e., perceived care; Q. 21)". Emotion ratings included: gratitude (Q. 9), indebtedness (Q. 10), guilt (Q. 11), and obligation (Q. 12). The questions for guilt [1-4] and obligation [5-9] were designed according to the operational definitions used by previous research. For events in which participants accepted help (Part 1), questions for behaviors included: "To what extent did you think you needed to reciprocate? (Q. 14)" The questions for other related factors (control variables) included: " How was your relationship with this benefactor before receiving help? (i.e., relationship with the benefactor; Q. 3)", "6. How helpful was the help? (i.e., the participant’s benefit; Q. 6)", and "7. How much was the benefactor's cost in this help? (i.e., the benefactor’s cost; Q. 7)". Questions were the same for Part 2 (i.e., events in which participants rejected help), except that participants were asked to *imagine* how they would feel or behave if they accepted this help.

**Word classification task.** An independent sample of participants was recruited (N = 80) to categorize the 100 words with the highest weight in the definitions of indebtedness collected in the large-scale online questionnaire in Study 1. They were instructed as following:

*In this experiment, you will see a list of words that people use in everyday helping and receiving helping situations. After seeing each word, please classify it into one of the following five categories according to your understanding:*

*1. Appraisal: if you think that the word is related to appraisals or evaluations on*

*the benefits, costs, intentions or other factors in the situation, please classify the word into this category.*

*2. Emotion (Feeling): if you think that this word is related to or reflects certain emotions and feelings of an individual in the situation, please classify the word into this category.*

*3. Behavior: if you think the word is related to the behavior of an individual in the situation, please classify the word into this category.*

*4. Person: if you think this word is related to person, please classify the word into this category.*

*5. Other: if you think the word has nothing to do with any of the four categories above, please classify the word into this category.*

***Study 2 - Interpersonal Task***

**Pain ratings.** In Study 2a and Study 2b, five to seven participants came to the experiment room together. An intra-epidermal needle electrode was attached to the left wrist of each participant for cutaneous electrical stimulation [10]. The first pain stimulation was set as 8 repeated pulses, each of which was 0.2 mA and lasted for 0.5 ms. A 10-ms interval was inserted between pulses. Then we gradually increased the intensity of each single pulse until the participant reported 6 on an 8-point pain scale (1 = not painful, 8 = intolerable). Participants reported that they would only experience the whole pulse train as a single stimulation, rather than as separate shocks. The final intensity of pain stimulation was calibrated to a subjective pain rating of "6", which was a moderate punishment for the participants.

**Experimental design for the interpersonal task.** Both Study 2a and Study 2b consisted of two sessions. All stimuli were presented using PsychToolBox 3.0.14

(www.psychtoolbox.org) in Matlab 2016a (Mathworks, Natick, MA, USA). Participants were instructed as following:

> *"In this experiment, you will play an interpersonal game, which is composed of two roles: the Decider and the Receiver. The Receiver will be in some trouble and the Decider can decide whether to help the Receiver at the cost of his/her own interests. Several previous participants have come to our lab during Stage 1 of our study and made decisions as the Deciders. Now this experiment belongs to Stage 2 of this study. In the two sessions of the experiment, you will perform as the Receiver, facing the decisions made by each anonymous previous Decider in Stage 1 and make your own decisions."*

Please see the main text for the methods of Session 1 (the main task).

In Study 2a, we manipulated the participant's beliefs about the benefactor's intentions (condition: Repayment possible vs. Repayment impossible) and benefactor's cost (12 levels of 5, 7, 8, 9, 10, 11, 12, 14, 15, 16, 18, 20) in a within-subject design. We included one trial for each condition-benefactor's cost combination for free-choice and force-accept situations, totaling 24 trials in each situation. As a result, there were a total of 48 trials with different anonymous co-players in Study 2a, which were randomized across participants.

Study 2b also included information about benefactor's intention and benefactor's cost. In addition, to disentangle the effect of the benefactor's cost and participant's benefit (i.e., pain reduction), we manipulated the exchange rate between the co-player's cost and participant's pain reduction (i.e., efficiency, 0.5, 1.0, and 1.5; the efficiency was always 1.0 in Study 2a) in a within-subject design. Thus, the participant's pain reduction was calculated as follows: Pain reduction = co-player's cost / co-player's

endowment × efficiency × maximum pain reduction (16s). In Study 2b, we included 5 levels for the benefactor's cost (i.e., 4, 8, 12, 16, 20). Participants were informed that the co-player could only choose discrete amounts of money to spend in this experiment. Furthermore, because the pain duration when benefactor's cost = 20 and efficiency = 1.5 exceeds the maximum pain reduction, this combination was eliminated, leaving 14 benefactor's cost-efficiency combinations. We included one trial for each condition-benefactor's cost-efficiency combination for free-choice and force-accept situations, totaling 28 trials in each situation. As a result, there were 56 trials with different anonymous co-players in Study 2b, which were randomized across participants. See Table S2 for more details about experimental settings.

**Post-task ratings.** In Session 2, the participant was shown the co-player's information (Information period with a blurred photo, co-player's ID and extra information about benefactor's intention, i.e., the benefactor knew Repayment possible or Repayment impossible; 4 sec) and their decision (Outcome period with benefactor's cost and the duration of pain reduction for the participant; 5 sec) of each trial from Session 1 in a random order. Then the participant was asked to recall and make the following ratings (from 0 to 100, with 0 represented "not at all" and 100 represented "extremely intense") at the time point when they had received the help of the co-player, but had not indicated decisions of accept/reject help and the amount of reciprocity.

- "How much gratitude do you feel for this co-player's decision?" (Gratitude)
- "How much indebtedness do you feel for this co-player's decision?" (Indebtedness)
- "How much do you think this decider cares about you?" (Perceived care)
- "How much pressure did you feel for the decider's expectation for repayment?" (Obligation)
- "How much guilt do you feel for this co-player's decision?"(Guilt)

The order of these ratings was counter-balanced across trials. The questions for self-reported ratings on guilt [1-4] and obligation [5-9] were based on previous research. Notably, the participant's second-order belief of how much the benefactor expected, decisions of whether to accept or reject help, and the amount of reciprocity were not shown in this session to minimize the influence of their prior behaviors on their reported feelings.

After the two sessions, five trials in Session 1 were randomly selected to be realized. The participant received the average pain stimulation in these five trials. The participant's final payoff was the average amount of endowment the participant left for him/herself across the chosen trials. The participant was instructed that the final payoff of each co-player was the amount of endowment the co-player left plus the amount of endowment the participant allocated to him/her. Participants were informed of this arrangement before the experiment began. After the experiment, participants were further debriefed that the co-players' decisions they were faced with during the experiment were actually pre-selected from participants' decisions in a previous experiment by the experimenters, and the co-players' decisions did not necessarily reflect the natural distributions of others' helping behaviors.

***Study 3 - FMRI Study***

The scanning session consisted of three runs (in total 54 trials) and lasted for approximately 39 min. Each run lasted for 13 min and consisted of 18 trials, including 9 levels of the benefactor's cost (4, 6, 8, 10, 12, 14, 16, 18, and 20) in Repayment possible condition and Repayment impossible conditions respectively. Trial order was randomized with each run. See Table S2 for additional details about the experimental design.

Each trial began with a 4 sec Information period, which showed the randomly selected

co-player's ID, blurred photo, and information of whether this co-player knew that the participant could or could not repay. This was followed by the 5 sec Outcome period, which included the co-player's decision on how much they spent to help the participant. Participants then had up to 8 sec to report how much he/she thought this co-player expected him/her to reciprocate (i.e., second-order belief of the co-player's expectation for repayment; rating scale from 0 to 25 using left and right buttons to move the cursor, step of 1 yuan). Next, participants had 8 sec to decide how much of their 25 yuan endowment (~ 3.4 USD) to reciprocate to the co-player (from 0 to 25 using left and right buttons to move the cursor, step of 1 yuan).

**Data analyses**

***Data Analyses in Study 1 (Online Questionnaire)***

Behavioral data analyses in Study 1 were carried out in IPython/Jupyter Notebook (Python 3.6.8) [11]. We used Wordcloud (version 1.8.0, https://amueller.github.io/word_cloud/index.html) and Jieba (version 0.42, https://github.com/fxsjy/jieba) packages to conduct text segmentation, and used collapsed Gibbs sampling implemented in lda package (version 1.1.0, https://lda.readthedocs.io/en/latest/) [12] to conduct Latent Dirichlet Allocation (LDA) based topic modeling on the emotional words of indebtedness. Results were plotted using matplotlib [13], and seaborn 0.9.0 (https://seaborn.pydata.org/index.html). All hypothesis tests are two-tailed.

**Validating Conceptual Model with Emotion Ratings.** All variables were normalized before the between-participant linear regressions predicting indebtedness ratings from guilt and obligation ratings. We additionally examined the degree of multicollinearity between guilt and obligation ratings using the variance inflation factor (VIF). The VIF reflects the degree that any regressor can be predicted by a linear combination of the other regressors (VIF = 5 serves as informal cutoff for multicollinearity, and lower numbers indicate less collinearity). Results demonstrated an acceptable level of multicollinearity between guilt and obligation ratings (Table S1). To rule out the possibility that these emotion ratings might covary with other related factors in Study 1 (e.g., benefactor's cost, the participant's benefit and the social distance between the participant and the benefactor), we re-estimated the above models with these factors as control variables, which did not appreciably change the results (Table S1).

**Validating Conceptual Model with Topic Modeling.** We used the Jieba package to process the text and excluded Chinese stopwords using the stopwords-json dataset (https://github.com/6/stopwords-json). Because Chinese retains its own characters of

various structures, we also combined synonyms of the same word as an additional preprocessing step [14]. Next, we computed a bag of words for each participant, which entailed counting the frequency that each participant used each word and transformed these frequencies using Term Frequency-Inverse Document Frequency (TF-IDF) [15,16]. This method calculates the importance of a word in the whole corpus based on the frequency of its occurrence in the text and the frequency of its occurrence in the whole corpus. The advantage of this method is that it can filter out some common but irrelevant words, while retaining important words that affect the whole text. Using this method, the 100 words with the highest weight/frequency in the definitions of indebtedness were extracted (Table S20). Words beyond these 100 had TF-IDF weights $< 0.01$ (Fig. S1b), indicating that the words included in the current analysis explained vast majority of variance in the definition of indebtedness. These 100 words were then classified by an independent sample of participants ($n = 80$) into levels of appraisal, emotion, behavior, person and other. We conducted Latent Dirichlet Allocation (LDA) based topic modeling on the emotional words of indebtedness using collapsed Gibbs sampling implemented in lda package. LDA is a generative probabilistic model for collections of discrete data such as text corpora, which is widely used to discover the topics that are present in a corpus [17]. It finds latent factors of semantic concepts based on the co-occurrence of words in participant's verbal descriptions without constraining participants' responses using rating scales, which currently dominates emotion research [18]. We compared the models with topic numbers ranging from 2 to 15 using 5-fold cross validation and found that the two-topic solution performed the best (Fig. S1c). Model goodness of fit was assessed using perplexity [19], which is a commonly used measurement in information theory to evaluate how well a statistical model describes a dataset, with lower perplexity denoting a better probabilistic model.

***Data analyses in Study 2 (Interpersonal Task)***

Behavioral data analyses in Studies 2 and 3 were carried out in RStudio version 1.1.383 [20]. Linear mixed models (LMMs) were conducted using lmeTest package (version 3.1-0) [21]. In Study 2, psych package (version 1.9.12) [22], nFactors package (version 2.4.1) [23], and GPArotation package (2014.11-1) [24] were used for the exploratory factor analysis (EFA). In Study 3, confirmatory factor analysis (CFA) was conducted using lavaan package [25]. In both Studies 2 and 3, computational modeling was carried out in Matlab 2016b (https://MathWorks.com) using CosanlabToolbox (https://github.com/ljchang/CosanlabToolbox). Results were plotted using matplotlib [13], and seaborn 0.9.0 (https://seaborn.pydata.org/index.html). All hypothesis tests are two-tailed.

**Validating Conceptual Model with Emotion Ratings.** Similar to Study 1, we tested whether guilt and obligation contribute to indebtedness using the trial-by-trial emotional ratings in Study 2. We fit LMMs predicting indebtedness ratings from guilt and obligation ratings with random intercepts and slopes for participants [26]. All variables were normalized before regression analysis. We additionally examined the degree of multicollinearity between guilt and obligation ratings using VIF (Table S1). To rule out the possibility that these emotion ratings might covary with other related factors, i.e., the experimental variables in Studies 2 (e.g., benefactor's cost, extra information about the benefactor’s intention and efficiency), we fit additional models controlling for these factors. Results of Study 2 replicated those in Study 1, and did not change after controlling for these variables (Table S1).

**The Effects of Experimental Manipulations on Participants' Appraisal, Emotional and Behavioral Responses.** All variables were normalized before the LMM analysis. Visualizations of the regression analyses (Fig. 4, b-c) were created using the lmplot function of seaborn in IPython/Jupyter Notebook and using the data of all trials for all

participants. We note that the standard error of means (SEMs) were generated via bootstrapping that respected the repeated measurements within a participant.

**Relationships between Appraisals and Emotions.** We estimated the correlations between appraisals (i.e., second-order belief and perceived care) and emotions (i.e., indebtedness, guilt, obligation, and gratitude) at both within-participant and between-participant levels. For within-participant analysis, for each pair of these six variables, we estimated the Pearson correlation for each participant, transformed the data using a fisher *r* to *z* transformation, and then conducted a one-sample *t*-test using *z* values of all participants to evaluate whether the two variables were significantly correlated at the group level (two-tailed, FDR corrected). This analysis captured the variability of appraisals and emotions across trials within participants (Fig. S3a, Table S8). For between-participant analysis, for each of the six variables, we computed the average value of the variable across all trials for each participant. We then estimated the correlations between each pair of variables based on variability across participants (two-tailed, FDR corrected; Fig. S3b, Table S9).

We then conducted a factor analysis to examine the relationship between appraisals and emotions [24]. The Kaiser-Meyer-Olkin (KMO) Measure of Sampling Adequacy [27] and Bartlett's test of sphericity [28] showed that the current data sets in Studies 2 and 3 were adequately sampled and met the criteria for factor analysis (Study 2: KMO value = 0.76, Bartlett's test $\chi^2 = 8801.85$, $df = 15$, $p < 0.001$; Study 3: KMO value = 0.77, Bartlett's test $\chi^2 = 2970.53$, $df = 15$, $p < 0.001$). All the variables were centered within participant to exclude the influences of individual differences in the range of ratings. We first applied EFA in Study 2 to identify the number of common factors and the relationships between appraisals and emotions. To determine the number of components to retain, the correlation matrix between the 6 variables was submitted to a parallel analysis using the psych package. Parallel analysis performs a principal factor decomposition of the

data matrix and compares it to a principal factor decomposition of a randomized data matrix. This analysis yields components whose eigenvalues (magnitudes) are greater in the observed data relative to the randomized data. The nScree function in nFactors package was used to determine the number of factors to retain. The result pointed to a two-factor solution (Fig. S2e). Factors were then estimated and extracted by combining ML factor analysis with oblique rotation using the GPArotation package. Next, we conducted CFA using the data of Study 3 to test the two-factor model built by Study 2 in an independent sample using the lavaan package. Results remained the same after controlling for the experimental variables.

Finally, we conducted a multivariate mediation analysis using structural equation modeling using the lavaan package. In this analysis, experimental variables (extra information about benefactor's intention, benefactor's cost, information-cost interaction, and efficiency) were taken as independent variables, ratings of second-order belief and perceived care were taken as mediators, and ratings of guilt, gratitude and the sense of obligation were taken as dependent variables. First, we built a full model that included all pathways between variables. Then, non-significant pathways in the full model were excluded from the full model to improve the fitness of the model. In the final model, experimental variables included extra information about the benefactor's intentions, the benefactor's cost, and their interaction; efficiency was excluded due to the non-significant effects. Moreover, in the final model, second-order beliefs mediated the effects of the experimental variables on obligation, whereas perceived care mediated the effects of experimental variables on guilt and gratitude. This model performed well (RSMEA = 0.023, SRMR = 0.004, CFI = 1.000, TLI = 0.997, BIC = 27496.99) and explained participants' responses better than the full model (RSMEA = 0.046, SRMR = 0.004, CFI = 1.000, TLI = 0.986, BIC = 27543.52).

**Using Communal and Obligation Factors as Predictors for Behaviors.** To investigate how participants' appraisals and emotions influenced their behavioral responses, we conducted two separate LMMs to predict participants' reciprocity decisions and help-acceptance decisions, respectively. Each model included the scores for Communal and Obligation Factors estimated from the factor analysis (Fig. 4d) as fixed effects and random intercepts and slopes for participants. All variables were normalized before regression analysis. See detailed results in the Supplementary Notes.

**Computational model fitting.**

*Model fitting for reciprocity decisions*

We estimated the model parameters for Model 1.1 by minimizing the sum of squared errors of the percentages that the model's behavioral predictions deviated from actual behaviors across all the trials that participants had to passively accept help [29]. Specifically, for each parameterization, we select the maximum $U(D_B)$ across the range of $D_B$ (i.e., $max(U(D_B))$) to predict each participant's reciprocity decision for each trial. We calculate the difference between the participant's actual choice and the model predicted choice, yielding SSE, or the residual sum of squares. We used Matlab's fmincon routine to identify parameters that minimized the sum of SSE of all trials separately for each participant.

$$SSE = \sum_{t=1}^{n} \left( \frac{D_B(t) - \max\big(U(D_B(t))\big)}{\gamma_B} * 100 \right)^2 \tag{13}$$

with $t$ indicating trial number. To avoid ending the fitting procedure at a local minimum, the model-fitting algorithm was initialized at 1000 random points in the three-dimensional theta-phi-kappa parameter space for each participant.

*Model fitting for help-acceptance decisions*

We computed the probability of the decision of whether to accept or reject help using a softmax specification with inverse temperature parameter *λ,* which ranges from [0,1]. In each trial, the probability of the participant choosing to accept help is given by

$$P(Accept) = \frac{e^{U(Accept)/\lambda}}{e^{U(Accept)/\lambda} + e^{U(Reject)/\lambda}} \tag{14}$$

We then conducted maximum likelihood estimation at the individual level by minimizing the negative log likelihood of the decision that the participant made ($D_B$ = Accept or Reject) over each trial *t* with 1000 different starting values across the four dimensional theta-phi-kappa-lambda parameter space using the fmincon routine implemented in Matlab:

$$LLE = -\sum_{t=1}^{n} \log\left(P(D_B(t))\right) \tag{15}$$

**Validations of Computational Modeling.**

*Model comparison for reciprocity decisions*

We compared Model 1.1 with other plausible models, including: (a) a model with linear formulations of utilities for self-interest, communal concern and obligation (Model 1.2), (b) models that solely included the term for communal concern (Model 1.3) or obligation (Model 1.4) besides the self-interest term, (c) models with separate parameters for self-interest, communal concern and obligation with separate parameters (Model 1.5 and Model 1.6), (d) a model that assumes participants reciprocate purely based on the benefactors helping behavior (i.e., tit-for-tat) (Model 1.7) [30,31], and (e) a model that assumes that participants are motivated to minimize inequity in payments

(Model 1.8) [29,32]. Model performance was measured and compared using the AIC [33], which rewards model fit and penalizes model complexity (number of free parameters).

First, the non-linear formulations in Model 1.1 are a standard way to model social preferences in utility functions [29,34] by adding convexity to the utility function via exponential error signals resulting from failing to meet perceived social standards (the benefactor's expectation or the benefactor's perceived care). In terms of model performance, this non-linear model for reciprocity outperformed a model with linear formulations of utilities for self-interest, communal concern, and obligation (Model 1.2; Tables S10, S11 and S14).

***Model 1.2:***

$$U(D_B) = \theta_B * (\gamma_B - D_B) - (1-\theta_B) * \left(\phi_B * max(\omega_B * \gamma_B - D_B, 0) + (1-\phi_B) * max(E_B'' - D_B, 0)\right)$$

To examine the necessities of both communal concern and obligation, we compared the full model (Model 1.1) to models that solely include term for communal concern (Model 1.3) or obligation (Model 1.4) besides the self-interest term.

***Model 1.3:***

$$U(D_B) = \theta_B * \frac{\gamma_B - D_B}{\gamma_B} - (1-\theta_B) * \left(\frac{\omega_B * \gamma_B - D_B}{\gamma_B}\right)^2$$

***Model 1.4:***

$$U(D_B) = \theta_B * \frac{\gamma_B - D_B}{\gamma_B} - (1-\theta_B) * \left(\frac{E_B'' - D_B}{\gamma_B}\right)^2$$

We further compared Model 1.1 to a model that independently parameterized the communal concern and obligation terms (Model 1.5), in which the ranges of parameters were defined as [0, 1].

***Model 1.5:***

$$U(D_B) = \theta_B * \frac{\gamma_B - D_B}{\gamma_B} - (1-\theta_B) * \left(\phi_{B1} * \left(\frac{\omega_B * \gamma_B - D_B}{\gamma_B}\right)^2 + \phi_{B2} * \left(\frac{E_B'' - D_B}{\gamma_B}\right)^2\right)$$

We also compared Model 1.1 to a model that independently parameterized the self-interest, communal concern and obligation terms (Model 1.6), in which the ranges of parameters were defined as [0, 1].

***Model 1.6:***

$$U(D_B) = \theta_B * \frac{\gamma_B - D_B}{\gamma_B} - \phi_{B1} * \left(\frac{\omega_B * \gamma_B - D_B}{\gamma_B}\right)^2 - \phi_{B2} * \left(\frac{E_B'' - D_B}{\gamma_B}\right)^2$$

Another possibility was that participants did not make decisions based on feelings of communal concern and obligation, but instead decided how much to reciprocate simply according to the benefactor's cost. The closer the amount of reciprocity to the benefactor's cost, the larger the utility of this action. This is similar to standard models of reciprocity [30,31]. In the Repayment impossible and Repayment possible conditions, the weights on the benefactor's cost in the total utility were different, captured by parameters $\phi_{B1}$ and $\phi_{B2}$ respectively, resulting in the behavioral differences under different conditions (Model 1.7). In this model, $C$ is the condition-indicating coefficient: $C = 1$ represents Repayment impossible condition, and $C = 0$ represents the Repayment possible condition.

***Model 1.7:***

$$U(D_B) = \theta_B * \frac{\gamma_B - D_B}{\gamma_B} + (1-\theta_B) * (C * \phi_{B1} * U_{Cost} + (1-C) * \phi_{B2} * U_{Cost})$$

$$U_{Cost} = -\left(\frac{D_A - D_B}{\gamma_B}\right)^2$$

The third possibility is that participants made decisions according to inequity aversion [32,35], which assumes that participants care not only about self-interest, but also the payoff absolute difference between self and other (Model 1.8). The smaller the difference between self-payoff and the benefactor's payoff (or the closer the reciprocity is to 1/2 of their total payoff), the greater the utility of this action [29,32]. In the Repayment impossible and Repayment possible conditions, the weights on inequity were different, captured by parameters $\phi_1$ and $\phi_2$ respectively, resulting in the behavioral differences under different conditions.

***Model 1.8***:

$$U(D_B) = \theta_B * \frac{\gamma_B - D_B}{\gamma_B} + (1-\theta_B) * \left(C * \phi_{B1} * U_{Inequity} + (1-C) * \phi_{B2} * U_{Inequity}\right)$$

$$U_{Inequity} = -\left(\frac{\gamma_B - D_B}{\gamma_A - D_A + D_B} - \frac{1}{2}\right)^2$$

*Model comparison for help-acceptance decisions*

We compared Model 2.1 with other plausible models, including: (a) models that solely included the term for communal concern (Model 2.2) or obligation (Model 2.3) besides the self-interest term, (c) models with separate parameters for self-interest, communal concern and obligation with separate parameters (Model 2.4 and Model 2.5). Model performance was measured and compared using the AIC [33]. In all the models, *U(Reject)* was set to zero, because the participant’s emotional responses would not change if the participant did not accept help.

***Model 2.2:***

$$U(Accept) = \theta_B * \frac{D_A * \mu}{\max(D_A * \mu)} + (1-\theta_B) * \omega_B$$

***Model 2.3:***

$$U(Accept) = \theta_B * \frac{D_A * \mu}{\max(D_A * \mu)} - (1 - \theta_B) * \frac{E_B^{"}}{\gamma_B}$$

We further compared the Model 2.1 to a model that had separate parameters for communal concern and obligation (Model 2.4), in which the ranges of $\theta$ and $\phi_2$ were defined as [0,1], and the ranges of $\phi_1$ was defined as [-1,1].

***Model 2.4***:

$$U(Accept) = \theta_B * \frac{D_A * \mu}{\max(D_A * \mu)} + (1 - \theta_B) * \left(\phi_{B1} * \omega_B - \phi_{B2} * \frac{E_B^{"}}{\gamma_B}\right)$$

We also compared Model 2.1 to a model that independently parameterized the self-interest, communal concern and obligation terms (Model 2.5), in which the ranges of $\theta$ and $\phi_2$ were defined as [0,1], and the ranges of $\phi_1$ was defined as [-1,1].

***Model 2.5***:

$$U(Accept) = \theta_B * \frac{D_A * \mu}{\max(D_A * \mu)} + \phi_{B1} * \omega_B - \phi_{B2} * \frac{E_B^{"}}{\gamma_B}$$

*Parameter recovery.*

Covariance between model terms implies that there might be multiple configurations of parameters that can produce the same predicted behavior. This means that, in practice, the more that these constructs covary, the less identifiable our parameters will become. We conducted parameter recovery analyses to ensure that our models were robustly identifiable [36]. To this end, we simulated data for each participant using our model parameters and the data from each trial of the experiment and compared how well we were able to recover these parameters by fitting the model to the simulated data. We

refit the model using 1000 random start locations to minimize the possibility of the algorithm getting stuck in a local minimum. We then assessed the degree to which the parameters could be recovered by calculating the similarity between all the parameters estimated from the observed behavioral data and all the parameters estimated from the simulated data using a Pearson correlation.

*Associations between model predictions and actual responses.* To validate the model representations of appraisals/feelings, we predicted participants self-reported appraisals, emotions and the two factors extracted from EFA separately using the trial-to-trial model representations of second-order beliefs $E_B''$ (Eq. 3) and perceived care $\omega_B$ (Eq. 5) in the reciprocity model by conducting LMMs that included random intercepts and slopes for each participant. All variables were normalized before regression analysis.

***Data analyses in Study 3 (FMRI Study)***

FMRI data preprocessing and univariate analyses were conducted using Statistical Parametric Mapping software SPM12 (Wellcome Trust Department of Cognitive Neurology, London). Meta-analytic decoding was conducted using the Neurosynth Image Decoder [37] (http://neurosynth.org). FMRI multivariate analyses were performed with our open source Python NLTools package [38] version 0.3.14 (https://nltools.org/). Results were plotted using matplotlib [13], and seaborn 0.9.0 (https://seaborn.pydata.org/index.html). All hypothesis tests are two-tailed.

**FMRI Data Acquisition and Preprocessing.** Images were acquired using a 3T Prisma Siemens scanner (Siemens AG, Erlangen, Germany) with a 64-channel head coil at Peking University (Beijing, China). T2-weighted echoplanar images (EPI) were obtained with blood oxygenation level-dependent (BOLD) contrast. Sixty-two transverse slices of 2.3 mm thickness that covered the whole brain were acquired using

multiband EPI sequence in an interleaved order (repetition time = 2000 ms, echo time = 30 ms, field of view = 224×224 $mm^2$, flip angle = 90°). Images were slice-time corrected, motion corrected, resampled to 3 mm × 3 mm × 3 mm isotropic voxels, and normalized to MNI space using the EPInorm approach in which functional images are aligned to an EPI template, which is then nonlinearly warped to stereotactic space [39]. Images were then spatially smoothed with an 8 mm FWHM Gaussian filter, and temporally filtered using a high-pass filter with a cutoff frequency of 1/128 Hz.

**Univariate fMRI Analyses.** Regressors for GLM1 of reciprocity and GLM 2 of communal concern included: (a) Outcome period (onset of the presentation of the benefactor's decision, 5s) with the corresponding parametric modulator, (b) Information period (onset of the presentation of the benefactor's photo and extra information regarding intention, 4s), (c) Second-order belief rating period (starting from the time the rating screen presented and spanning to the time that the participant made choice), (d) Allocation period (starting from the time the rating screen presented and spanning to the time that the participant made choice), (e) Missed responses (the missing decision period for second-order belief or allocation, 8s), and (f) six head motion realignment parameters. Contrasts were defined as the positive effect of the parametric modulator of interest.

For GLM3 of obligation, because our computational model's representation of second order beliefs $E_B''$ had a non-normal distribution (zero in Repayment impossible condition and linear increase in Repayment possible condition, Eq. 3), we constructed a piecewise linear contrast, instead of linear parametric analysis. This entailed creating four separate regressors modeling different parts of the function during the Outcome period: (1) Repayment impossible, (2) Repayment possible and low benefactor's cost (i.e., 4, 6, or 8), (3) Repayment possible and medium benefactor's cost (i.e., 10, 12, or 14), (4) Repayment possible and high benefactor's cost (i.e., 16, 18, or 20).

Subsequently, for each participant, we constructed a contrast vector of c = [-6, 1, 2, 3]. This piecewise linear contrast ensures that brain responses to the Repayment impossible trials are lower than all of the Repayment possible trials. We have successfully used this approach in previous work modeling guilt using similar Psychological Game Theoretic utility models [40].

To be noted, in GLMs for communal concern and obligation, we chose to use the terms of $\omega_B$ and $E_B''$ rather than the $U_{Communal}$ and the $U_{Obligation}$ terms, as the latter ones create costs based on the squared deviation from reciprocity behavior, which results in a large proportion of trials where the deviations are near zero as a result of participant's decisions, making them inefficient for parametric analysis to capture how successfully participants behaved in accordance with their feelings. Instead, $\omega_B$ and $E_B''$ better captured the inferences that comprised participants' feelings and were more suitable for testing our hypotheses about brain responses.

For all GLMs, events in each regressor were convolved with a double gamma canonical hemodynamic response function. Second-level models were constructed as one-sample $t$ tests using contrast images from the first-level models. For whole brain analyses, all results were corrected for multiple comparisons using cluster correction $p < 0.05$ with a cluster-forming threshold of $p < 0.001$, which attempts to control for family wise error (FWE) using Gaussian Random Field Theory. This approach attempts to estimate the number of independent spatial resels or resolution elements in the data necessary to control for FWE. This calculation requires defining an initial threshold to determine the Euler Characteristic of the data. It has been demonstrated that an initial threshold of $p < 0.001$ does a reasonable job of controlling for false positives at 5% using this approach [41].

**Meta-analytical Decoding.** Using the Neurosynth Image Decoder [37], we evaluated the spatial similarity between the unthresholded contrast maps of reciprocity, communal concern and obligation with the reverse inference meta-analytical maps for 23 terms generated from this database, related to basic cognition (i.e., Imagine, Switching, Salience, Conflict, Memory, Attention, Cognitive control, Inhibition, Emotion, Anxiety, Fear, and Default mode) [42], social cognition (Empathy, Theory of mind, Social, and Imitation) [43] and decision-making (Reward, Punishment, Learning, Prediction error, Choice, and Outcome) [44].

**Neural Utility Model of Indebtedness.**

*Multivariate whole-brain models.* We trained two separate multivariate whole-brain models predictive of communal concern ($\omega_B$) and obligation ($E_B''$) terms in our behavioral model separately for each participant using principal components regression with 5-fold cross-validation [45-47]. This entailed first performing temporal data reduction by estimating single-trial beta maps of the Outcome period for each participant. Then for each participant, we separately predicted $\omega_B$ and $E_B''$ from a vectorized representation of the single trial beta maps. Because these models have considerably more voxel features (~328k) then trial observations, we performed a principal components analysis to reduce the feature space and used the principal components to predict the model appraisal representations (e.g., $\omega_B$ and $E_B''$). We then back-projected the estimated beta components from the regression back into the full voxel feature space, and then back to 3-D space. We have previously demonstrated that this approach is effective in reliably mapping the independent contribution of each voxel in the brain to a psychological state to identify the neural representations of affective states [46,48,49]. We estimated the performance of each whole-brain model by extracting the cross-validated prediction accuracy ($r$ value) for each participant, conducting $r$-to-$z$ transformation, and then conducting a one-sample permutation $t$-test.

As a benchmark for our neural utility model, we were interested in determining how well we could predict participant's reciprocity behavior directly from brain activity. This benchmark model was trained using the same training procedure described above, but predicted trial-to-trial reciprocity behavior separately for each participant. In theory, this should provide a theoretical upper bound of the best we should be able to predict reciprocity behavior using brain activity. If our neural utility model is close, then it means that we are able to predict reciprocity behavior using brain representations of communal concern and obligation as well as the optimal linear weighting of brain weights that can predict trial-to-trial reciprocity behavior.

To determine the importance of the participant-specific model parameters in the neural utility model, we ran a permutation test to determine how well we could predict reciprocity behavior for each participant using parameters from a randomly selected different participant. We ran 5,000 permutations to generate a null distribution of average prediction accuracy after randomly shuffling the participant weights. The empirical $p$-value is the proportion of permutations that exceed our average observed correlation.

*Relative spatial alignment.* The intuition for the relative pattern similarity analysis (Eq. 12) is that if the optimal brain map for predicting a participant's decision is relatively more similar to their communal concern or obligation map, then we would expect that the participant cared more about that particular component of indebtedness during behavioral decision-making. For example, if a participant weights obligation more than communal concern during reciprocity (higher $1 - \phi$ estimated from the behavioral model), then the spatial similarity between their obligation brain pattern and the pattern that directly predicts their reciprocity behavior (reciprocity brain pattern) should be relatively higher compared to the spatial similarity between their communal concern pattern and reciprocity brain pattern.

**Online questionnaire for Study 1**

(Note: The questions included in final analysis are labeled in bold.)

*Welcome to participate in this questionnaire survey! In the questionnaire, please recall your real life events, and answer the corresponding questions. After answering all the questions, please write a short story about each event. Your story may be used as material for future study. If your story is selected, we will contact you through your contact information and pay you 25 yuan for story authorization. When using the story, we will keep your personal information strictly confidential. Please fill in the answer sheet sincerely and carefully.*

*To ensure the quality of the data, please check the following box, and promise that you will have at least 15 minutes to conduct the survey and answer each question sincerely. Thank you for your cooperation!*

○ I guarantee that I have at least 15 minutes to fill out the questionnaire and answer each question sincerely.

**Part 1**

Have you received any help in the past one year? [single choice]

○ Yes (Continue)　○ No (skip Part 1)

Please think carefully about an event in which you received help from others that impressed you the most in the past one year and happened recently.

1. What is the time of this event? (Please select the option that matches the occurrence of the event and is closest to today)

○Within one week ○Within one month,

○Within three months ○Within half a year ○Within one year

2. In this event, did you actively seek help or passively accept help from others? [single choice]

○Actively seek　○Passive acceptance

**3. How was your relationship with this benefactor before receiving help? (0 is very unfamiliar, 100 is very familiar)**

4. To what extent were you willing to accept this help? (0 is not willing to accept, 100

very willing to accept)

5. Who was the person that helped you? [single choice]

○Parent ○Sibling ○Spouse/boyfriend/girlfriend ○Other relative ○Friend

○Classmate/Colleague ○Teacher ○Neighbor ○Stranger

○Other (such as service personnel, public servants, etc.. Please fill in the benefactor's specific occupation) _________________

**6. How helpful was the help? (0 is useless, 100 is very helpful)**

**7. How much was the benefactor's cost in this help? (0 is not at all, 100 is very big)**

8. Before receiving the help, how likely did you think the benefactor would help? (0 is completely impossible, 100 is pretty sure)

**9. How grateful did you feel about the benefactor's help? (0 is not at all, 100 is very strong)**

**10. How indebted did you feel about the benefactor's help? (0 is not at all, 100 is very strong)**

**11. How guilty did you feel about the benefactor's help? (0 is not at all, 100 is very strong)**

**12. How much were you afraid of the benefactor's expectation for repay? (0 is not at all, 100 is very strong)**

13. How much pressure did you feel to reciprocate in the future? (0 is not at all, 100 is very strong)

**14. To what extent did you think you needed to reciprocate? (0 is not at all, 100 is very strong)**

**15. To what extent do you think the benefactor expected you to repay? (0 is not at all, 100 is very strong)**

16. Compared with the benefit you obtained from the help, how much did you think you needed to reciprocate to the benefactor? (0-50 means less than your benefit, 50 means equal to your benefit, 50-100 means more than your benefit)

17. Compared with the benefactor's cost, how much did you think you needed to reciprocate to the benefactor? (0-50 means less than the benefactor's cost, 50 means equal to the benefactor's cost, 50-100 means more than the benefactor's cost)

18. Compared with the benefit you obtained from the help, how much did you think the benefactor needed you to reciprocate? (0-50 means less than your benefit, 50 means

equal to your benefit, 50-100 means more than your benefit)

19. Compared with the benefactor's cost, how much did you think the benefactor needed you to reciprocate? (0-50 means less than the benefactor's cost, 50 means equal to the benefactor's cost, 50-100 means more than the benefactor's cost)

20. Did the benefactor propose a clear request for reciprocity? [single choice]

○ Yes, please briefly explain the details _________________ ○ No

**21. To what extent did you think the benefactor cared about your welfare when helping you? (0 is not at all, 100 is very strong)**

22. To what extent did you think the benefactor cared about his/her own interests when helping you? (0 is not at all, 100 is very strong)

23. To what extent did you want to reciprocate? (0 is not at all, 100 is very strong)

24. To what extent did you want to repay the favor immediately? (0 is not at all, 100 is very strong)

25. In what way did you want to reciprocate? [multiple choice]

□ Monetary reciprocity □ Help each other

□ Oral thanks □ Establish cooperative relationship

□ Make friends with him/her □ Gifts □ Other _________________

26. Have you reciprocated in some way? [multiple choice]

□ Monetary reciprocity □ Help each other

□ Oral thanks □ Establish cooperative relationship

□ Make friends with him/her □ Gifts □ Other _________________

27. To what extent were you willing to interact or get to know each other further? (0 is not at all, 100 is very strong)

28. Please describe the event in detail in the form of a short story.

(If your story is selected as the material for our experiment, you will receive a story authorization fee of 25 yuan)

**Part 2**

Have you rejected any help in the past one-year? [single choice]

○ Yes (Continue) ○ No (skip Part 2)

Please think carefully about an event when you received help from others that

impressed you most in the past one year and happened recently.

1. What is the time of this event? (Please select the option that matches the occurrence of the event and is closest to today)

○Within one week ○Within one month,

○Within three months ○Within half a year ○Within one year

2. In this event, did the benefactor actively provide the offer of help or did someone suggest the benefactor to give you help? [single choice]

○Actively provided the offer of help ○Someone suggested the benefactor to help

**3. How was your relationship with this benefactor before this event? (0 is very unfamiliar, 100 is very familiar)**

4. To what extent did you want to reject the offer of help? (0 is not at all, 100 is very strong)

5. Who was the person that offered to help you? [single choice]

○Parent ○Sibling ○Spouse/boyfriend/girlfriend ○Other relative ○Friend

○Classmate/Colleague ○Teacher ○Neighbor ○Stranger

○Other (such as service personnel, public servants, etc. Please fill in the benefactor's specific occupation) _________________

**6. Imagine if you have accepted the help, how helpful would the help be? (0 is useless, 100 is very helpful)**

**7. Imagine if you have accepted the help, how much would the benefactor's cost be in this help? (0 is not at all, 100 is very big)**

8. Before receiving the help, how likely did you think the benefactor would help? (0 is completely impossible, 100 is pretty sure)

**9. Imagine if you have accepted the help, how grateful would you feel about the benefactor's help? (0 is not at all, 100 is very strong)**

**10. Imagine if you have accepted the help, how indebted would you feel about the benefactor's help? (0 is not at all, 100 is very strong)**

**11. Imagine if you have accepted the help, how guilty would you feel about the benefactor's help? (0 is not at all, 100 is very strong)**

**12. Imagine if you have accepted the help, how much you were afraid of the**

**benefactor's expectation for repay? (0 is not at all, 100 is very strong)**

13. Imagine if you have accepted the help, how much pressure would you feel to reciprocate in the future? (0 is not at all, 100 is very strong)

**14. Imagine if you have accepted the help, to what extent did you think you needed to reciprocate? (0 is not at all, 100 is very strong)**

**15. Imagine if you have accepted the help, to what extent would you think the benefactor expected you to reciprocate? (0 is not at all, 100 is very strong)**

16. Imagine if you have accepted the help, compared with the benefit you obtained from the help, how much would you think you needed to reciprocate to the benefactor? (0-50 means less than your benefit, 50 means equal to your benefit, 50-100 means more than your benefit)

17. Imagine if you have accepted the help, compared with the benefactor's cost, how much would you think you needed to reciprocate to the benefactor? (0-50 means less than the benefactor's cost, 50 means equal to the benefactor's cost, 50-100 means more than the benefactor's cost)

18. Imagine if you have accepted the help, compared with the benefit you obtained from the help, how much would you think the benefactor needed you to reciprocate? (0-50 means less than your benefit, 50 means equal to your benefit, 50-100 means more than your benefit)

19. Imagine if you have accepted the help, compared with the benefactor's cost, how much would you think the benefactor needed you to reciprocate? (0-50 means less than the benefactor's cost, 50 means equal to the benefactor's cost, 50-100 means more than the benefactor's cost)

20. Did the benefactor ask for repayment before helping you? [single choice]

○Yes, please briefly explain the details _________________ ○No

**21. To what extent did you think the benefactor cared about your welfare when he/she offered to help you? (0 is not at all, 100 is very strong)**

22. To what extent did you think the benefactor cared about his/her own interests when he/she offered to help you? (0 is not at all, 100 is very strong)

23. Imagine if you have accepted the help, to what extent would you want to reciprocate? (0 is not at all, 100 is very strong)

24. Imagine if you have accepted the help, to what extent did you want to repay the favor immediately? (0 is not at all, 100 is very strong)

25. Imagine if you have accepted the help, in what way did you want to reciprocate? [multiple choice]

□ Monetary reciprocity □ Help each other

□ Oral thanks □ Establish cooperative relationship

□ Make friends with him/her □ Gifts □ Other

27. What was/were your reason(s) for refusing the offer? [multiple choice]

□ Thought the benefactor's purpose not pure

□ The anticipatory repayment was too much

□ Limit your freedom

□ Feeling your self-esteem was hurt

□ The benefit from the help was little

□ The benefactor's cost was too much

28. Please describe the event in detail in the form of a short story.

(If your story is selected as the material for our experiment, you will receive a story authorization fee of 25 yuan)

**• In the context of helping and receiving help, what is your definition of gratitude?**

**• In the context of helping and receiving help, what is your definition of indebtedness?**

**• In daily life, what do you think is/are the source(s) of indebtedness? (Single choice, the order of the first two options was counterbalanced among participants)**

**□ Negative feeling for harming the benefactor/for cost that the benefactor has paid for helping you**

**☐ Negative feeling for the pressure to repay caused by other's ulterior intentions (e.g., Expectation for repay)**

**☐ Both of the above**

**☐ Neither of the above**

## Supplementary Notes

### Predicting behaviors using Communal and Obligation Factors

Because our computational model cannot differentiate between appraisals and feelings, we also report the results predicting behavior using the factors combining these ratings estimated from the exploratory factor analysis (EFA; Fig, 4d). We conducted linear mixed model for reciprocity by including the scores for Communal Factor and Obligation Factor extracted from EFA as fixed effects with by-participant random slopes for each fixed effect. Results demonstrated that both factors contributed significantly to reciprocity (Communal Factor: $\beta = 0.58 \pm 0.03$, 95%CI = [0.52, 0.64], $t(90.04) = 17.20$, $p < 0.001$; Obligation Factor: $\beta = 0.20 \pm 0.02$, 95%CI = [0.16, 0.24], $t(80.54) = 9.21$, $p < 0.001$). Similarly, both communal and obligation factors contributed significantly to the decisions of whether to accept help (Communal Factor: $\beta = -0.13 \pm 0.04$, 95%CI = [-0.21, -0.05], $t(88.46) = -17.55$, $p < 0.001$; Obligation Factor: $\beta = 0.06 \pm 0.03$, 95%CI = [0.00, 0.12], $t(91.97) = 7.76$, $p < 0.001$). These results are consistent with separately predicting behavior from appraisals and emotions as reported in the main text.

### Results of efficiency manipulation

In Study 2b, we further manipulated the participant's benefit from help by varying the exchange rate between the co-player's cost and participant's pain reduction (i.e., **Efficiency**, 0.5, 1, and 1.5) on the basis of Study 2a where Efficiency was 1. The higher the Efficiency, the more benefit the participant would obtain from each amount of the co-player's cost. Results are presented in Table S6. Specifically, first, in line with previous studies on gratitude showing that the beneficiary's benefit contributes positively to the feeling of gratitude [50-52], we found that the higher the Efficiency (i.e., the more benefit the participant obtained), the higher the participant's self-reported feeling of gratitude ($\beta = 0.05 \pm 0.02$, 95%CI = [0.01, 0.09], $t(67.60) = 3.27$, $p < 0.001$).

Similarly, participants' self-reported ratings of indebtedness were positively correlated with the size of Efficiency ($\beta = 0.04 \pm 0.01$, 95%CI = [0.02, 0.06], $t(67.35) = 2.65$, $p = 0.008$). In contrary, the Efficiency did not contribute significantly to participants' rating of guilt and the sense of obligation (guilt: $\beta = 0.01 \pm 0.01$, 95%CI = [-0.0.1, 0.03], $t(60.12) = 0.74$, $p = 0.458$; obligation: $\beta = 0.00 \pm 0.02$, 95%CI = [-0.04, 0.04], $t(58.64) = -0.03$, $p = 0.975$) (Table S6).

To be noted, since the effects of Efficiency (or the benefit the beneficiary obtained from help) on beneficiary's emotions are not the focus of the current study, the range of Efficiency was relatively small in the current study (i.e., 0.5, 1, 1.5). Therefore, it is possible that the current manipulation was not efficient to capture some relatively extreme situations, which resulted in the current non-significant effects of Efficiency on guilt and the sense of obligation. For example, will a beneficiary feel less guilt when the benefactor's small cost has a larger effect, or feel more guilt when the benefactor's large cost has a small effect? Future specially designed studies are needed to explore how the efficiency of help influences the beneficiary's emotional responses.

## Supplementary References


1 Benedict, R. Chrysanthemum and the Sword. Patterns of Japanese Culture, Cleveland, New York (The World Publishing Company) 1946. (1946).

2 Kotani, M. Expressing gratitude and indebtedness: Japanese speakers' use of "I'm sorry" in English conversation. *Res. Lang. Soc. Interac.* **35**, 39-72 (2002).

3 Naito, T. & Washizu, N. Note on cultural universals and variations of gratitude from an East Asian point of view. *J. Behav. Sci.* **10**, 1-8 (2015).

4 Washizu, N. & Naito, T. The emotions sumanai, gratitude, and indebtedness, and their relations to interpersonal orientation and psychological well-being among Japanese university students. *International Perspectives in Psychology: Research, Practice, Consultation* **4**, 209 (2015).

5 Greenberg, M. S. in *Social exchange* 3-26 (Springer, 1980).

6 Greenberg, M. S. & Westcott, D. R. Indebtedness as a mediator of reactions to aid. *New directions in helping* **1**, 85-112 (1983).

7 Naito, T. & Sakata, Y. Gratitude, Indebtedness, and Regret on Receiving a Friend's Favor in Japan. *Psychologia* **53**, 179-194 (2010).

8 Tsang, J. A. The effects of helper intention on gratitude and indebtedness. *Motiv. Emotion* **30**, 199-205 (2006).

9 Watkins, P. C., Scheer, J., Ovnicek, M. & Kolts, R. The debt of gratitude: Dissociating gratitude and indebtedness. *Cognition Emotion* **20**, 217-241 (2006).

10 Inui, K., Tran, T. D., Hoshiyama, M. & Kakigi, R. Preferential stimulation of Adelta fibers by intra-epidermal needle electrode in humans. *Pain* **96**, 247-252 (2002).

11 Pérez, F. & Granger, B. E. IPython: a system for interactive scientific computing. *Comput. Sci. Eng.* **9** (2007).

12 Blei, D. M., Ng, A. Y. & Jordan, M. I. Latent dirichlet allocation. *J. Mach. Learn. Res.* **3**, 993-1022 (2003).

13 Hunter & John, D. Matplotlib: A 2D Graphics Environment. *Comput. Sci. Eng.* **9**, 90-95 (2007).

14 Liu, Q. in *International Conference on Computer Science & Network Technology.* (2016).

15 Neto, J. L., Santos, A. D., Kaestner, C. A., Alexandre, N. & Santos, D. Document clustering and text summarization. (2000).

16 Salton, G. & Buckley, C. Term-weighting approaches in automatic text retrieval. *Inform. Process. Manag.* **24**, 513-523 (1988).

17 Blei, D. M. & Lafferty, J. D. in *Proceedings of the 23rd international conference on Machine learning.* 113-120 (ACM).

18 Cowen, A. S. & Keltner, D. Self-report captures 27 distinct categories of emotion bridged by continuous gradients. *Proceedings of the National Academy of Sciences* **114**, E7900 (2017).

19 Blei, D. M., Ng, A. Y. & Jordan, M. I. Latent dirichlet allocation. *J. Mach. Learn. Res.* **3**, 993–1022 (2003).

20 Racine, J. S. RStudio: A Platform‐Independent IDE for R and Sweave. *J. Appl. Economet.* **27**, 167-172 (2012).

21 Kuznetsova, A., Brockhoff, P. B. & Christensen, R. H. lmerTest package: tests in linear mixed effects models. *J. Stat. Softw.* **82**, 1-26 (2017).

22 Revelle, W. An overview of the psych package. *Department of Psychology Northwestern University. Accessed on March* **3**, 2012 (2011).

23 Raiche, G., Magis, D. & Raiche, M. G. Package ‘nFactors’. *Repository CRAN*, 1-58 (2020).

24 Fabrigar, L. R., Wegener, D. T., MacCallum, R. C. & Strahan, E. J. Evaluating the use of exploratory factor analysis in psychological research. *Psychol. Methods* **4**, 272-299 (1999).

25 Rosseel, Y. lavaan: An R package for structural equation modeling. *J. Stat. Softw.* **48**, 1-36 (2012).

26 Barr, D. J., Levy, R., Scheepers, C. & Tily, H. J. Random effects structure for confirmatory hypothesis testing: Keep it maximal. *Journal of memory and language* **68**, 255-278 (2013).

27 Hair, J. F., Black, W. C., Babin, B. J., Anderson, R. E. & Tatham, R. *Multivariate data analysis*. (Uppersaddle River, 2006).

28 Tobias, S. & Carlson, J. E. Brief report: Bartlett's test of sphericity and chance findings in factor analysis. *Multivar. Behav. Res.* **4**, 375-377 (1969).

29 van Baar, J. M., Chang, L. J. & Sanfey, A. G. The computational and neural substrates of moral strategies in social decision-making. *Nat. Commun.* **10**, 1-14 (2019).

30 Dufwenberg, M. & Kirchsteiger, G. A theory of sequential reciprocity. *Game. Econ. Behav.* **47**, 268-298 (2004).

31 Rabin, M. Incorporating fairness into game theory and economics. *Am. Econ. Rev.*, 1281-1302 (1993).

32 Fehr, E. & Schmidt, K. M. A theory of fairness, competition, and cooperation. *Q. J. Econ.* **114**, 817-868 (1999).

33 Akaike, H. A new look at the statistical model identification. *IEEE transactions on automatic control* **19**, 716-723 (1974).

34 Bolton, G. E. & Ockenfels, A. ERC: A theory of equity, reciprocity, and competition. *Am. Econ. Rev.* **90**, 166-193 (2000).

35 Bolton, G. E. & Ockenfels, A. ERC: A theory of equity, reciprocity, and competition. *Am. Econ. Rev.*, 166-193 (2000).

36 Fareri, D. S., Chang, L. J. & Delgado, M. R. Computational substrates of social value in interpersonal collaboration. *J. Neurosci.* **35**, 8170-8180 (2015).

37 Yarkoni, T., Poldrack, R. A., Nichols, T. E., Van Essen, D. C. & Wager, T. D. Large-scale automated synthesis of human functional neuroimaging data. *Nature Methods* **8**, 665 (2011).

38 Chang, L. J., Jolly, E., Cheong, J.H., Burnashev, A., Chen, P.Y.A., Clark, M.,

Frey, S., & Fitzpatrick, P. cosanlab/nltools: 0.4.7 (v0.4.7). *Zenodo* https://doi.org/10.5281/zenodo.7015135 (2022).

39 Calhoun, V. D. *et al.* The impact of T1 versus EPI spatial normalization templates for fMRI data analyses. *Hum. Brain Mapp.* **38**, 5331-5342 (2017).

40 Chang, L. J., Smith, A., Dufwenberg, M. & Sanfey, A. G. Triangulating the neural, psychological, and economic bases of guilt aversion. *Neuron* **70**, 560-572 (2011).

41 Woo, C.-W., Krishnan, A. & Wager, T. D. Cluster-extent based thresholding in fMRI analyses: Pitfalls and recommendations. *Neuroimage* **91**, 412-419 (2014).

42 Barrett, L. F. & Satpute, A. B. Large-scale brain networks in affective and social neuroscience: towards an integrative functional architecture of the brain. *Curr. Opin. Neurobiol.* **23**, 361-372 (2013).

43 Adolphs, R. The social brain: neural basis of social knowledge. *Annu. Rev. Psychol.* **60**, 693-716 (2009).

44 Ruff, C. C. & Fehr, E. The neurobiology of rewards and values in social decision making. *Nat. Rev. Neurosci.* **15**, 549 (2014).

45 Woo, C. W., Chang, L. J., Lindquist, M. A. & Wager, T. D. Building better biomarkers: brain models in translational neuroimaging. *Nat. Neurosci.* **20**, 365-377 (2017).

46 Chang, L. J., Gianaros, P. J., Manuck, S. B., Krishnan, A. & Wager, T. D. A sensitive and specific neural signature for picture-induced negative affect. *PLoS Biol.* **13**, e1002180 (2015).

47 Wager, T. D. *et al.* An fMRI-based neurologic signature of physical pain. *N. Engl. J. Med.* **368**, 1388-1397 (2013).

48 Chang, L. J. *et al.* Endogenous variation in ventromedial prefrontal cortex state dynamics during naturalistic viewing reflects affective experience. *Science Advances* **7**, eabf7129 (2021).

49 Woo, C.-W., Chang, L. J., Lindquist, M. A. & Wager, T. D. Building better

biomarkers: brain models in translational neuroimaging. *Nat. Neurosci.* **20**, 365-377 (2017).

50 Tesser, A., Gatewood, R. & Driver, M. Some determinants of gratitude. *J. Pers. Soc. Psychol.* **9**, 233 (1968).

51 Yu, H., Gao, X., Zhou, Y. & Zhou, X. Decomposing gratitude: representation and integration of cognitive antecedents of gratitude in the brain. *J. Neurosci.*, 2944-2917 (2018).

52 Elfers, J. & Hlava, P. *The Spectrum of Gratitude Experience*. (Springer, 2016).

## Supplementary Figures

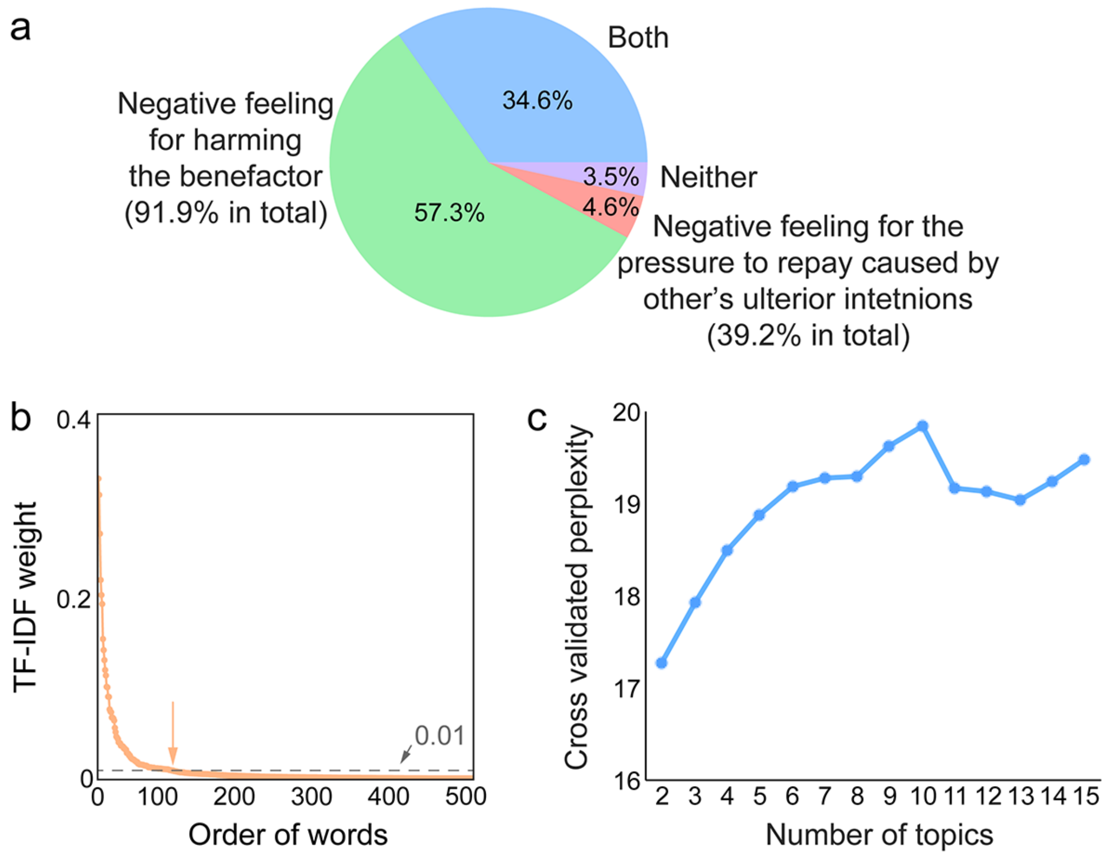


**Fig. S1 | Definition of indebtedness. a** The frequency of choosing each option in the question "In daily life, what do you think is/ are the source(s) of indebtedness?" (n = 1619 participants) While 57.3% of participants indicated the negative feeling for harming the benefactor as the single source of indebtedness, 4.6% of participants indicated the negative feeling for the pressure of repayment caused by other's ulterior intentions as the single source of indebtedness. 34.6% of participants indicated both types of negative emotions contributing to indebtedness, and 3.5% of participants indicated neither of them as the source of indebtedness. **b** Emotional words were extracted from the 100 words with the highest weight/frequency in the definitions of indebtedness based on the annotation by an independent sample of participants (n = 80). Note, words beyond these 100 had TF-IDF weights < 0.01, indicating that the words included in the current analysis explained vast majority of variance in the definition of indebtedness. **c** We selected the best number of topics by comparing the models with topic numbers ranging from 2 to 15 using 5-folds cross validation. Model goodness of fit was assessed using perplexity, with lower perplexity denoting a better probabilistic model. We found that the two-topic solution performed the best.

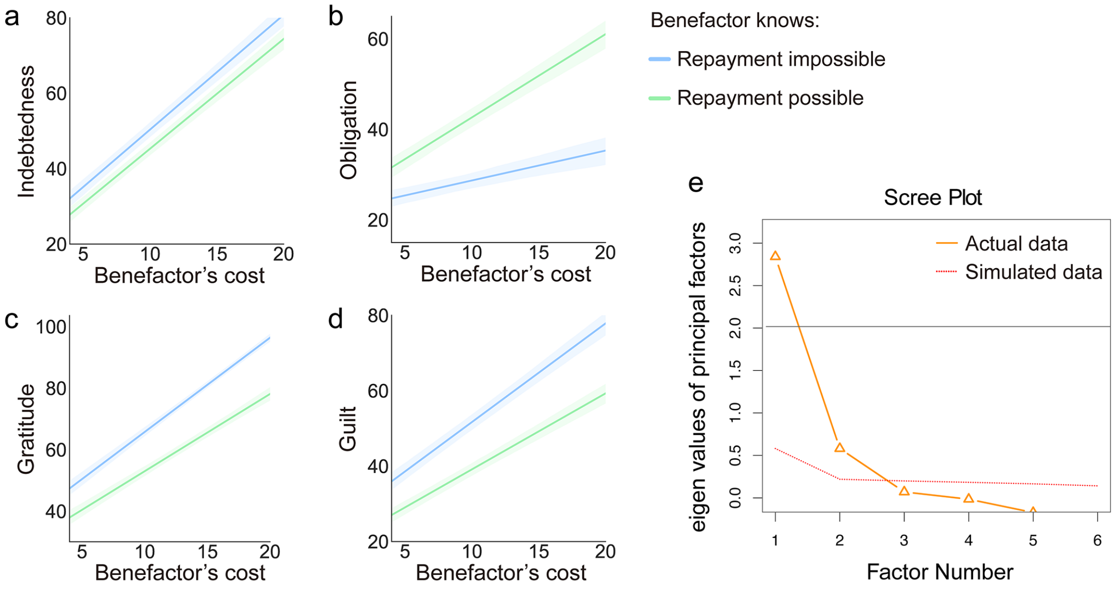


**Fig. S2 | a-d** Participant's ratings on indebtedness, obligation, gratitude and guilt plotted as functions of the extra information about benefactor's intention and benefactor's cost in Study 2. Data are presented as mean values +/- SEM. SEMs were generated via bootstrapping that respected the repeated measurements within a participant (n = 24 trials for each condition in Study 2a/28 trials for each condition in Study 2b, 108 participants). **e** To determine the number of factors to retain in the exploratory factor analysis (EFA) in Study 2 (n = 108 participants), the correlation matrix between appraisals and emotions was submitted to a parallel analysis [14]. Parallel analysis performed a principal factor decomposition of the data matrix and compared it to a principal factor decomposition of a randomized data matrix. This analysis yielded factors whose eigenvalues (magnitudes) were greater in the observed data relative to the randomized data. The nScree function in nFactors package was used to return an analysis of the number of factors to retain. The result pointed to a two-factor solution except for the acceleration factor.

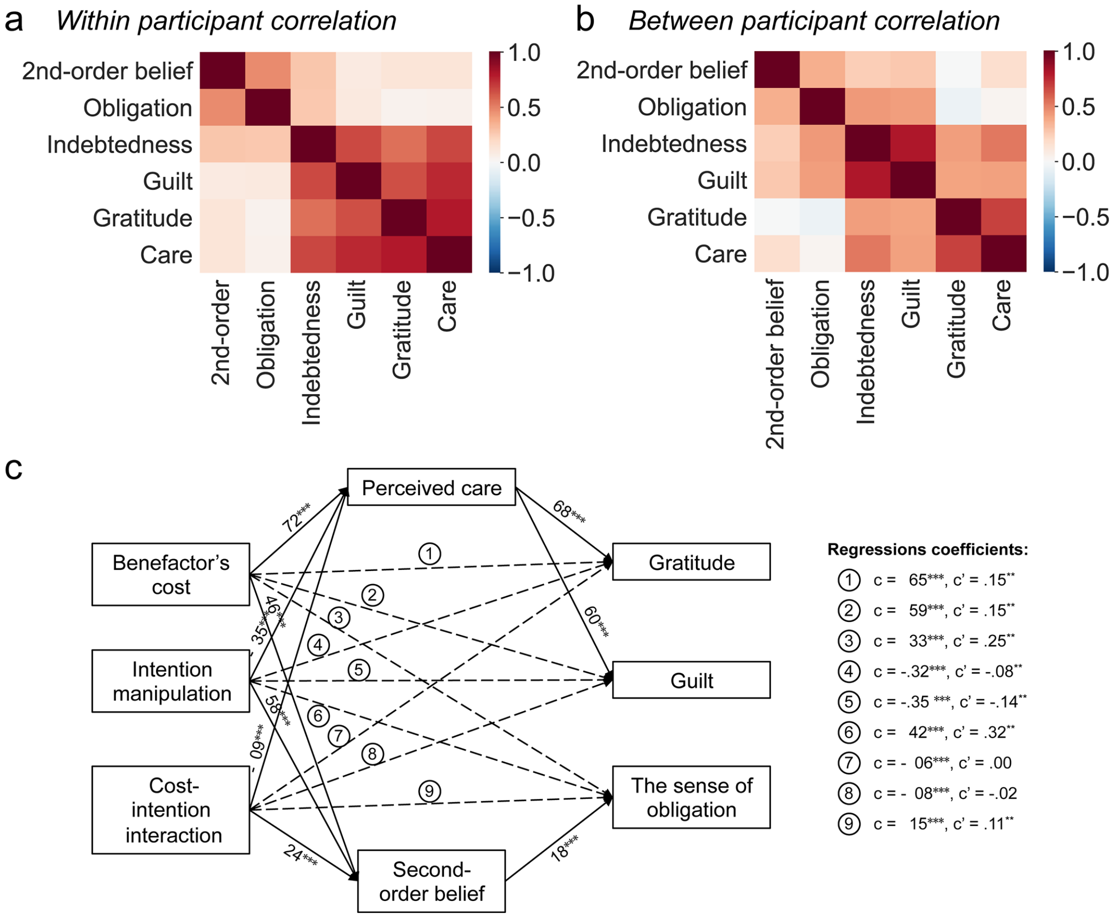


**Fig. S3 | Relationships between appraisals and emotions in Study 2 (n = 108 participants). a-b** Correlation matrix between participant's appraisal and emotion ratings built based on within participant (a) and between participant (b) variances, respectively. **c** Mediation analysis showed that second-order beliefs and perceived care appraisals differentially mediated the effects of the experimental manipulations on emotional responses (total indirect effect = 0.59±0.04, 95%CI = [0.51, 0.67], $Z$ = 14.49, $p$ < 0.001, two-tailed; model performance: $\chi^2$ = 9.68, $df$ = 4, CFI =1.00, TLI = 0.997, RMSEA = 0.023, SRMR = 0.004; Fig. 4e and Fig. S3c). Second-order beliefs mediated the effects of the experimental manipulations on obligation (Indirect effect = 0.22±0.03, 95%CI = [0.16, 0.29], $Z$ = 7.18, $p$ < 0.001), while perceived care mediated the effects of the experimental manipulations on guilt (Indirect effect = 0.17±0.01, 95%CI = [0.15, 0.20], $Z$ = 13.23, $p$ < 0.001) and gratitude (Indirect effect = 0.19±0.01, 95%CI = [0.17, 0.22], $Z$ = 13.72, $p$ < 0.001).

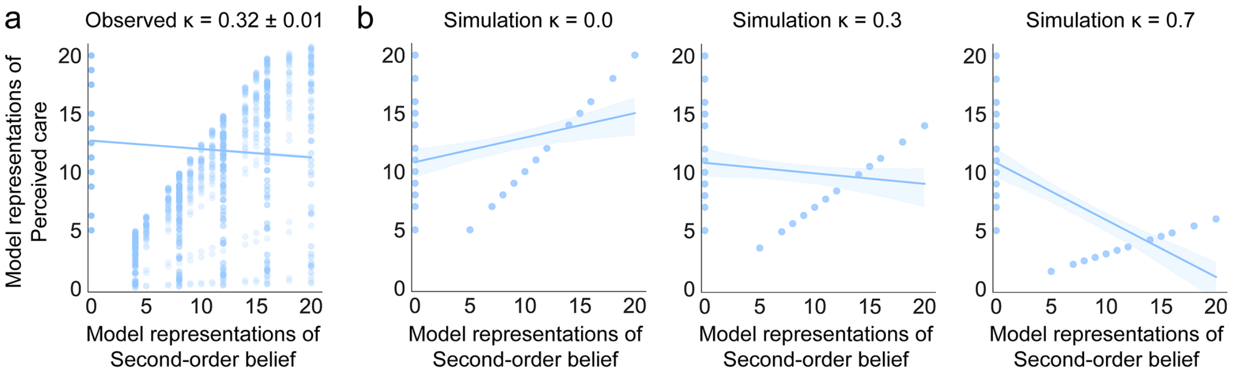


**Fig. S4 | The relationship between model predicted second-order belief and perceived care. a** Relationships between model representations of second-order belief $E_B''$ and perceived care $\omega_B$ in Study 2. Data are presented as mean values +/- SEM. SEMs were generated via bootstrapping that respected the repeated measurements within a participant (n = 24 trials for each condition in Study 2a/28 trials for each condition in Study 2b, 108 participants). **b** The simulated data with different levels of $\kappa$. Data are presented as mean values +/- SEM.

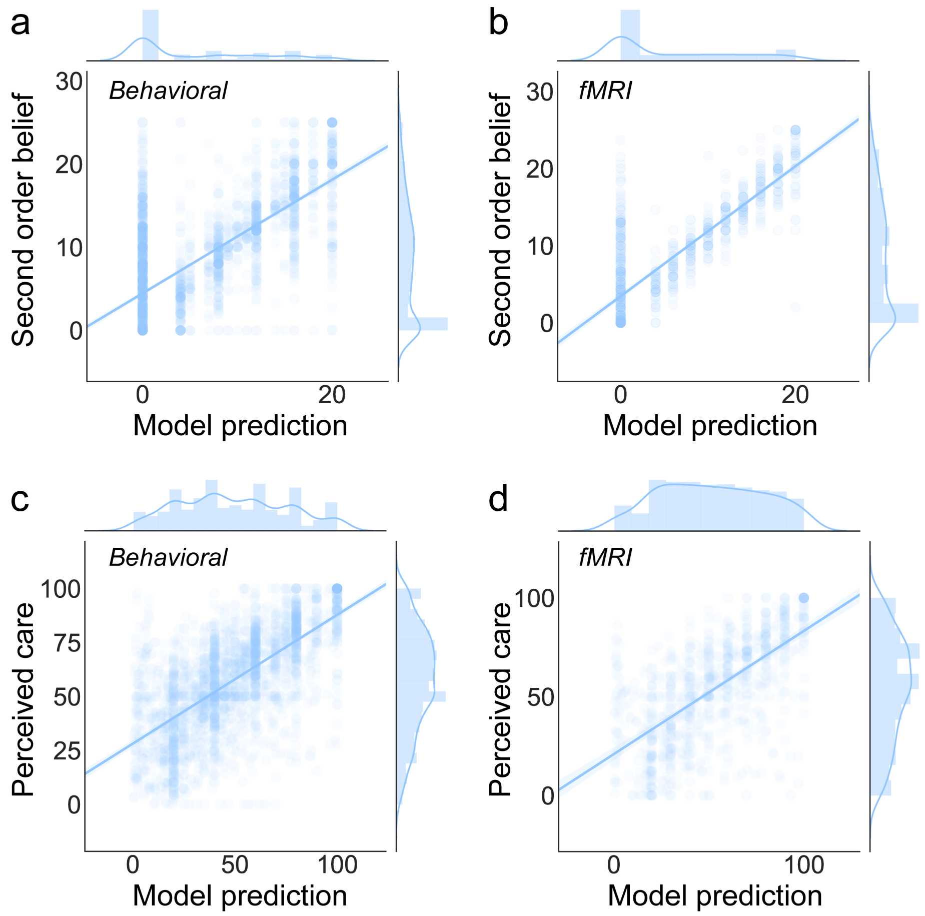


**Fig. S5 | Model predictions for appraisals on benefactor's intentions.** Regression analyses showed that the reciprocity model's representations of $E_B''$ and $\omega_B$ were associated with trial-to-trial variations in self-reported values of second-order belief of the benefactor's expectation for repayment ($\beta_{behavioral}$ = 0.68 ± 0.03 (mean ± SE), 95%CI = [0.62, 0.74], $t(106.93) = 21.48$, $p < 0.001$; $\beta_{fMRI}$ = 0.78 ± 0.04, 95%CI = [0.71, 0.86], $t(52.05) = 21.89$, $p < 0.001$, linear mixed model, two-tailed, a and b) and perceived care ($\beta_{behavioral}$ = 0.72 ± 0.03, 95%CI = [0.66, 0.77], $t(107.30) = 26.76$, $p <$ 0.001; $\beta_{fMRI}$ = 0.74 ± 0.04, 95%CI = [0.66, 0.82], $t(52.66) = 18.13$, $p < 0.001$, c and d), which provided further validation that the model representations were reflecting the intended psychological processes. Data are presented as mean values +/- SEM. SEMs were generated via bootstrapping that respected the repeated measurements within a participant. For a and c, n = 24 trials for each condition in Study 2a/28 trials for each condition in Study 2b over 108 participants. For b and d, n = 27 trials for each condition over 53 participants.

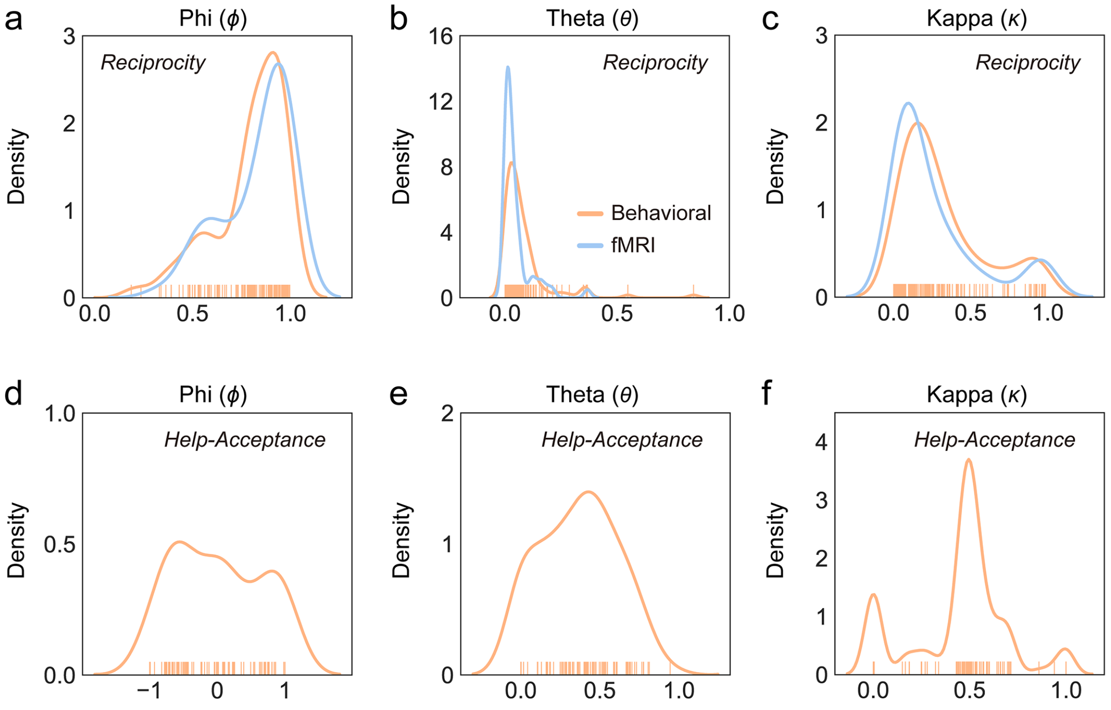


**Fig. S6 | Distributions of model parameters. a-c** Distributions of parameters for reciprocity decisions in behavioral (Study 2, n = 108 participants) and fMRI (Study 3, n = 53 participants) studies. **d-f** Distributions of parameters for help-acceptance decisions in the behavioral study (Study 2, n = 108 participants).

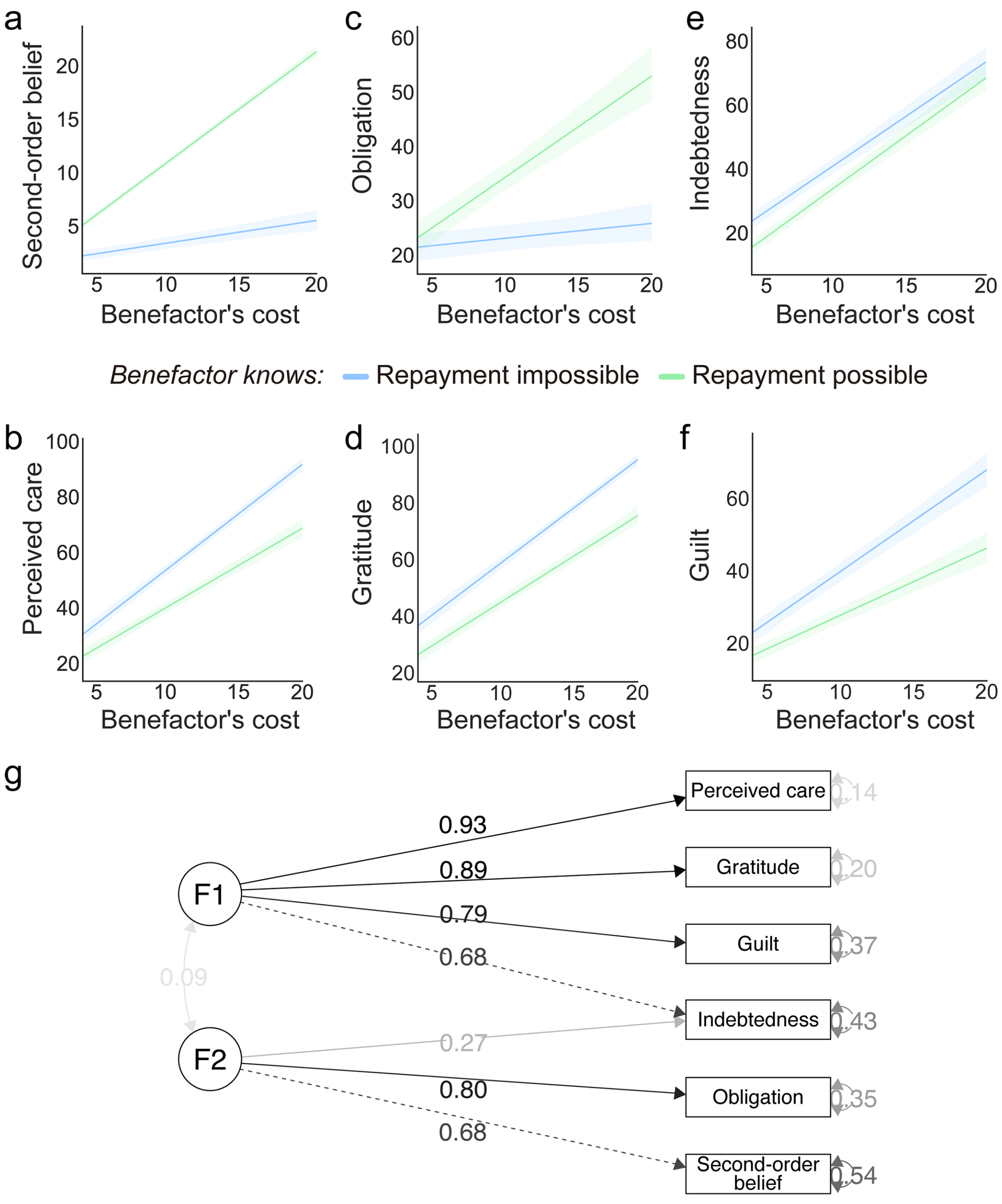


**Fig. S7 | Appraisal and emotional responses in the fMRI study (Study 3, n = 53 participants) replicated that in the behavioral studies.** Participant's ratings of second-order belief of the benefactor's expectation for repay (**a**), perceived care (**b**), sense of obligation (**c**), gratitude (**d**), indebtedness (**e**) and guilt (**f**) plotted as functions of extra information about benefactor's intention (Repayment impossible vs. Repayment possible) and benefactor's cost. Data are presented as mean values +/- SEM. SEMs were generated via bootstrapping that respected the repeated

measurements within a participant (n = 27 trials for each condition over 53 participants). **g** We conducted confirmatory factor analysis (CFA) in Study 3 to test the two-factor model (Fig. 4e) built by Study 2 in an independent sample. Results showed that the fitness of this two-factor model is appropriate ($\chi^2$ = 48.33, *df* = 7, RSMEA = 0.079, SRMR = 0.019, CFI = 0.986, TLI = 0.970).

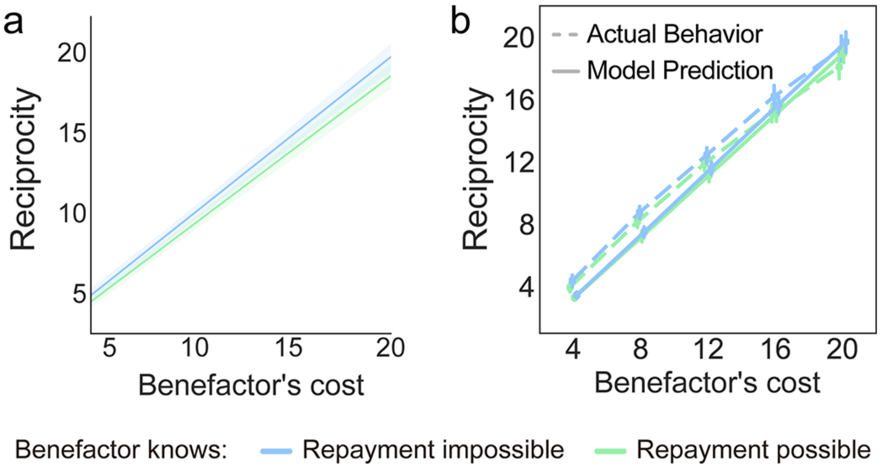


**Fig. S8 | The results of reciprocity in the fMRI study (Study 3, n = 53 participants) replicates that in the behavioral studies (Study 2). a** Participant's amount of reciprocity plotted as functions of extra information about benefactor's intention (Repayment impossible vs. Repayment possible) and benefactor's cost. Data are presented as mean values +/- SEM. SEMs were generated via bootstrapping that respected the repeated measurements within a participant (n = 27 trials for each condition, 53 participants). **b** Our computational model accurately captured the patterns of participants' reciprocity after receiving help, $r^2$ = 0.95, $\beta$ = 0.91±0.02, 95%CI = [0.88, 0.94], $t(40.97)$ = 57.87, $p$ < 0.001, linear mixed model, two-taild. Data are presented as mean values +/- SEM.

## Supplementary Tables

**Table S1. The contributions of guilt and obligation ratings to indebtedness ratings**

| | Model | Predictor | Df | AIC | Term | Beta | SE | 95%CI | *t* | *df* | $p_{FDR}$ |
|---|---|---|---|---|---|---|---|---|---|---|---|
| **Study 1** | | | Before controlling for benefactor's cost, participant's benefit and social distance | | | | | | | | |
| | | | ***Model 3 vs. Model 1***: $F$ = 1606.10, $p$ < 0.001; | | | | | | | | |
| | | | ***Model 3 vs. Model 2***: $F$ = 5.34, $p$ = 0.021; VIF = 1.22. | | | | | | | | |
| | Model 1 | Obligation | 3 | 5412.8 | Obligation | 0.34 | 0.02 | [0.30, 0.38] | 16.05 | 1989 | < 0.001 |
| | Model 2 | Guilt | 3 | 4239.1 | Guilt | 0.71 | 0.02 | [0.68, 0.74] | 45.41 | 1989 | < 0.001 |
| | **Model 3** | Obligation | 4 | 4235.8 | Obligation | 0.40 | 0.02 | [0.36, 0.44] | 2.31 | 1988 | 0.021 |
| | | + Guilt | | | Guilt | 0.70 | 0.02 | [0.66, 0.73] | 40.08 | 1988 | < 0.001 |
| | | | | | After controlling for benefactor's cost, participant's benefit and social distance | | | | | | |
| | | | | | ***Model 3 vs. Model 1***: $F$ = 1005.70, $p$ < 0.001; | | | | | | |
| | | | | | ***Model 3 vs. Model 2***: $F$ = 3.53, $p$ = 0.060; VIF = 1.14. | | | | | | |
| | Model 1 | Obligation | 3 | 19004.3 | Obligation | 0.42 | 0.03 | [0.37, 0.47] | 15.87 | 1989 | < 0.001 |
| | Model 2 | Guilt | 3 | 17913.3 | Guilt | 0.74 | 0.02 | [0.70, 0.77] | 43.44 | 1989 | < 0.001 |
| | **Model 3** | Obligation | 4 | 17907.6 | Obligation | 0.61 | 0.02 | [0.57, 0.65] | 2.77 | 1988 | 0.005 |
| | | + Guilt | | | Guilt | 0.71 | 0.02 | [0.68, 0.75] | 38.26 | 1988 | < 0.001 |
| **Study 2** | | Before controlling for experimental variables | | | | | | | | | |
| | | ***Model 3 vs. Model 1***: $\chi^2$ = 1557.70, *df* = 4, $p$ < 0.001; | | | | | | | | | |
| | | ***Model 3 vs. Model 2***: $\chi^2$ = 599.69, *df* = 4, $p$ < 0.001; VIF = 1.44. | | | | | | | | | |
| | Model 1 | Obligation | 6 | 6270.1 | Obligation | 0.35 | 0.05 | [0.25, 0.46] | 6.51 | 106.48 | < 0.001 |
| | Model 2 | Guilt | 6 | 5312.0 | Guilt | 0.75 | 0.03 | [0.68, 0.81] | 22.54 | 97.88 | < 0.001 |
| | **Model 3** | Obligation | 10 | 4720.3 | Obligation | 0.27 | 0.03 | [0.20, 0.33] | 8.04 | 100.80 | < 0.001 |
| | | + Guilt | | | Guilt | 0.68 | 0.04 | [0.61, 0.75] | 19.36 | 97.78 | < 0.001 |
| | | After controlling for experimental variables | | | | | | | | | |
| | | ***Model 3 vs. Model 1***: $\chi^2$ = 245.44, *df* = 4, $p$ < 0.001; | | | | | | | | | |
| | | ***Model 3 vs. Model 2***: $\chi^2$ = 97.871, *df* = 4, $p$ < 0.001; VIF = 1.03. | | | | | | | | | |
| | Model 1 | Obligation | 6 | 7744.6 | Obligation | 0.22 | 0.04 | [0.15, 0.28] | 6.50 | 97.89 | < 0.001 |

| | Model | Predictor | Df | AIC | Term | Beta | SE | 95%CI | *t* | *df* | $p_{FDR}$ |
|---|---|---|---|---|---|---|---|---|---|---|---|
| | Model 2 | Guilt | 6 | 7590.1 | Guilt | 0.34 | 0.03 | [0.28, 0.39] | 11.30 | 99.81 | < 0.001 |
| | **Model 3** | Obligation | 10 | 7503.1 | Obligation | 0.17 | 0.03 | [0.12, 0.22] | 10.23 | 85.85 | < 0.001 |
| | | + Guilt | | | Guilt | 0.30 | 0.03 | [0.23, 0.35] | 6.24 | 94.45 | < 0.001 |
| **Study 3** | | Before controlling for experimental variables | | | | | | | | | |
| | | ***Model 3 vs. Model 1***: $\chi^2 = 462.43$, $df = 4$, $p < 0.001$; | | | | | | | | | |
| | | ***Model 3 vs. Model 2***: $\chi^2 = 128.47$, $df = 4$, $p < 0.001$; VIF = 1.98. | | | | | | | | | |
| | Model 1 | Obligation | 6 | 2319.2 | Obligation | 0.29 | 0.07 | [0.16, 0.43] | 4.31 | 47.44 | < 0.001 |
| | Model 2 | Guilt | 6 | 1985.2 | Guilt | 0.63 | 0.04 | [0.56, 0.71] | 15.83 | 44.47 | < 0.001 |
| | **Model 3** | Obligation | 10 | 1864.8 | Obligation | 0.20 | 0.05 | [0.10, 0.30] | 3.82 | 36.93 | < 0.001 |
| | | + Guilt | | | Guilt | 0.62 | 0.06 | [0.52, 0.70] | 13.64 | 38.27 | < 0.001 |
| | | After controlling for experimental variables | | | | | | | | | |
| | | ***Model 3 vs. Model 1***: $\chi^2 = 43.78$, $df = 4$, $p < 0.001$; | | | | | | | | | |
| | | ***Model 3 vs. Model 2***: $\chi^2 = 17.25$, $df = 4$, $p = 0.002$; VIF = 1.00. | | | | | | | | | |
| | Model 1 | Obligation | 6 | 2685.2 | Obligation | 0.17 | 0.04 | [0.09, 0.24] | 4.36 | 27.78 | < 0.001 |
| | Model 2 | Guilt | 6 | 2658.8 | Guilt | 0.22 | 0.04 | [0.14, 0.31] | 5.33 | 38.81 | < 0.001 |
| | **Model 3** | Obligation | 10 | 2649.6 | Obligation | 0.13 | 0.04 | [0.06, 0.20] | 3.70 | 31.58 | < 0.001 |
| | | + Guilt | | | Guilt | 0.20 | 0.04 | [0.12, 0.28] | 5.02 | 34.50 | < 0.001 |

Note: Data analyses were conducted using linear regression in Study 1, and using linear mixed models in Studies 2 and 3, two-tailed, FDR corrected.

**Table S2. Experimental designs for Study 2 and Study 3**

| Study | Study 2a | Study 2b | Study 3 |
|---|---|---|---|
| **Sample size** | 51 | 57 | 53 |
| **Conditions** | Benefactor knows: Repayment impossible vs. Repayment possible | Benefactor knows: Repayment impossible vs. Repayment possible | Benefactor knows: Repayment impossible vs. Repayment possible |
| **Cost** | 5, 7, 8, 9, 10, 11, 12,14, 15, 16, 18, 20 | 4, 8, 12, 16, 20 | 4, 6, 8, 10, 12, 14, 16, 18, 20 |
| **Efficiency manipulation** | 1 | 0.5, 1, 1.5 | 1 |
| **Dependent variables** | Reciprocity, Accept/Reject Help | Reciprocity, Accept/Reject Help | Reciprocity, |
| **Trial number of each cost-efficiency combination in each condition** | 1 | 1 | 3 |
| **Total trial number** | 48 | 56 | 54 |

**Table S3. The effects of the extra information about benefactor's intention, benefactor's cost, and efficiency on participants' emotional and behavioral responses (combining data of Studies 2a and 2b)**

| Dependent | Predictors | Beta | SE | 95%CI | *t* (*z*) | *df* | *p* |
|---|---|---|---|---|---|---|---|
| **Second-order belief** | Benefactor's cost | 0.42 | 0.02 | [0.38, 0.46] | 20.84 | 106.20 | < 0.001 |
| | Extra information about benefactor's intention | 0.53 | 0.03 | [0.47, 0.59] | 15.71 | 107.04 | < 0.001 |
| | Benefactor's cost×Extra information | 0.22 | 0.02 | [0.18, 0.26] | 13.13 | 103.83 | < 0.001 |
| **Perceived care** | Benefactor's cost | 0.63 | 0.03 | [0.57, 0.69] | 23.70 | 106.82 | < 0.001 |
| | Extra information about benefactor's intention | -0.31 | 0.02 | [-0.35, -0.27] | -13.89 | 106.44 | < 0.001 |
| | Benefactor's cost×Extra information | -0.08 | 0.01 | [-0.10, -0.06] | -6.64 | 98.59 | < 0.001 |
| **Gratitude** | Benefactor's cost | 0.55 | 0.03 | [0.49, 0.61] | 19.36 | 106.97 | < 0.001 |
| | Extra information about benefactor's intention | -0.27 | 0.02 | [-0.31, -0.23] | -13.18 | 106.35 | < 0.001 |
| | Benefactor's cost×Extra information | -0.06 | 0.01 | [-0.08, -0.04] | -4.20 | 99.31 | < 0.001 |
| **Indebtedness** | Benefactor's cost | 0.52 | 0.03 | [0.46, 0.58] | 20.24 | 106.29 | < 0.001 |
| | Extra information about benefactor's intention | -0.09 | 0.03 | [-0.15, -0.03] | -2.98 | 105.81 | 0.003 |
| | Benefactor's cost×Extra information | -0.01 | 0.01 | [-0.03, 0.01] | -0.72 | 92.52 | 0.474 |
| **Guilt** | Benefactor's cost | 0.41 | 0.02 | [0.37, 0.45] | 17.04 | 107.47 | < 0.001 |
| | Extra information about benefactor's intention | -0.25 | 0.02 | [-0.29, -0.21] | -10.30 | 106.30 | < 0.001 |
| | Benefactor's cost×Extra information | -0.05 | 0.01 | [-0.07, -0.03] | -4.28 | 96.94 | < 0.001 |
| **Obligation** | Benefactor's cost | 0.22 | 0.03 | [0.16, 0.28] | 7.71 | 107.03 | < 0.001 |
| | Extra information about benefactor's intention | 0.30 | 0.03 | [0.24, 0.36] | 9.28 | 106.82 | < 0.001 |
| | Benefactor's cost×Extra information | 0.11 | 0.01 | [0.09, 0.13] | 8.85 | 100.74 | < 0.001 |
| **Reciprocity** | Benefactor's cost | 0.63 | 0.02 | [0.59, 0.67] | 25.60 | 109.12 | < 0.001 |
| | Extra information about benefactor's intention | -0.05 | 0.02 | [-0.09, -0.01] | -3.30 | 106.99 | 0.001 |
| | Benefactor's cost×Extra information | -0.03 | 0.01 | [-0.05, -0.01] | -2.99 | 130.29 | 0.003 |
| **Decision to reject help** | Benefactor's cost | -0.65 | 0.13 | [-0.90, -0.40] | -5.16 | 2788 | < 0.001 |
| | Extra information about benefactor's intention | 0.27 | 0.08 | [0.11, 0.43] | 3.64 | 2788 | < 0.001 |
| | Benefactor's cost×Extra information | 0.07 | 0.07 | [-0.07, 0.21] | 1.08 | 2788 | 0.279 |

Note: Each analysis was carried out using linear mixed model, two-tailed, uncorrected, with independent hypotheses. Each hypothesis testing should have no association with raising the false positive rate of a separate hypothesis.

**Table S4. The effects of the extra information about benefactor's intention and benefactor's cost on participants' emotional and behavioral responses (Study 2a)**

| Dependent variable | Predictors | Beta | SE | 95%CI | *t* (*z*) | *df* | *p* |
|---|---|---|---|---|---|---|---|
| **Second-order belief** | Benefactor's cost | 0.37 | 0.03 | [0.31, 0.43] | 13.78 | 50.22 | < 0.001 |
| | Extra information about benefactor’s intention | 0.56 | 0.05 | [0.46, 0.66] | 11.41 | 50.00 | < 0.001 |
| | Benefactor’s cost×Extra information | 0.18 | 0.02 | [0.14, 0.22] | 7.38 | 50.04 | < 0.001 |
| **Perceived care** | Benefactor's cost | 0.51 | 0.04 | [0.43, 0.59] | 14.57 | 50.07 | < 0.001 |
| | Extra information about benefactor’s intention | -0.38 | 0.04 | [-0.46, -0.30] | -9.80 | 50.06 | < 0.001 |
| | Benefactor’s cost×Extra information | -0.08 | 0.02 | [-0.12, -0.04] | -3.99 | 49.75 | < 0.001 |
| **Gratitude** | Benefactor's cost | 0.49 | 0.04 | [0.41, 0.57] | 12.64 | 50.00 | < 0.001 |
| | Extra information about benefactor’s intention | -0.34 | 0.04 | [-0.42, -0.26] | -9.35 | 50.12 | < 0.001 |
| | Benefactor’s cost×Extra information | -0.05 | 0.02 | [-0.09, -0.01] | -2.54 | 50.08 | 0.014 |
| **Indebtedness** | Benefactor’s cost | 0.43 | 0.03 | [0.37, 0.49] | 12.31 | 50.10 | < 0.001 |
| | Extra information about benefactor’s intention | -0.07 | 0.06 | [-0.19, 0.05] | -1.10 | 50.00 | 0.277 |
| | Benefactor’s cost×Extra information | 0.00 | 0.02 | [-0.04, 0.04] | 0.08 | 50.29 | 0.935 |
| **Guilt** | Benefactor's cost | 0.33 | 0.03 | [0.27, 0.39] | 11.26 | 50.70 | < 0.001 |
| | Extra information about benefactor’s intention | -0.27 | 0.04 | [-0.35, -0.19] | -6.46 | 50.00 | < 0.001 |
| | Benefactor’s cost×Extra information | -0.30 | 0.02 | [-0.34, -0.26] | -1.56 | 50.35 | 0.126 |
| **Obligation** | Benefactor’s cost | 0.13 | 0.04 | [0.05, 0.21] | 3.67 | 50.06 | < 0.001 |
| | Extra information about benefactor’s intention | 0.36 | 0.05 | [0.26, 0.46] | 6.91 | 49.95 | < 0.001 |
| | Benefactor’s cost×Extra information | 0.17 | 0.02 | [0.13, 0.21] | 6.22 | 50.23 | < 0.001 |
| **Reciprocity** | Benefactor’s cost | 0.64 | 0.03 | [0.58, 0.70] | 22.92 | 50.11 | < 0.001 |
| | Extra information about benefactor’s intention | -0.05 | 0.03 | [-0.11, 0.01] | -1.66 | 50.45 | 0.103 |
| | Benefactor’s cost×Extra information | -0.03 | 0.02 | [-0.07, 0.01] | -1.47 | 64.43 | 0.147 |
| **Decision to reject help** | Benefactor’s cost | -0.37 | 0.21 | [-0.78, 0.04] | -1.70 | 1195 | 0.088 |
| | Extra information about benefactor’s intention | 0.30 | 0.11 | [0.08, 0.52] | 2.62 | 1195 | 0.009 |
| | Benefactor’s cost×Extra information | 0.14 | 0.11 | [-0.08, 0.36] | 1.28 | 1195 | 0.201 |

Note: Each analysis was carried out using linear mixed model, two-tailed, uncorrected, with independent hypotheses. Each hypothesis testing should have no association with raising the false positive rate of a separate hypothesis.

**Table S5. The effects of the extra information about benefactor's intention, and benefactor's cost on participants' emotional and behavioral responses (Study 2b)**

| Dependent variable | Predictors | Beta | SE | 95%CI | *t* (*z*) | *df* | *p* |
|---|---|---|---|---|---|---|---|
| **Second-order belief** | Benefactor's cost | 0.46 | 0.03 | [0.40, 0.52] | 15.63 | 56.01 | < 0.001 |
| | Extra information about benefactor’s intention | 0.50 | 0.05 | [0.40, 0.60] | 10.76 | 56.00 | < 0.001 |
| | Benefactor’s cost×Extra information | 0.25 | 0.02 | [0.21, 0.29] | 10.91 | 56.00 | < 0.001 |
| **Perceived care** | Benefactor's cost | 0.72 | 0.04 | [0.64, 0.80] | 19.49 | 55.99 | < 0.001 |
| | Extra information about benefactor’s intention | -0.25 | 0.02 | [-0.29, -0.21] | -10.55 | 55.95 | < 0.001 |
| | Benefactor’s cost×Extra information | -0.08 | 0.01 | [-0.10, -0.06] | -5.34 | 55.94 | < 0.001 |
| **Gratitude** | Benefactor's cost | 0.59 | 0.04 | [0.51, 0.67] | 14.71 | 56.00 | < 0.001 |
| | Extra information about benefactor’s intention | -0.22 | 0.02 | [-0.26, -0.18] | -9.54 | 55.81 | < 0.001 |
| | Benefactor’s cost×Extra information | -0.05 | 0.02 | [-0.09, -0.01] | -3.27 | 55.89 | < 0.001 |
| **Indebtedness** | Benefactor's cost | 0.59 | 0.04 | [0.51, 0.67] | 16.27 | 56.00 | < 0.001 |
| | Extra information about benefactor’s intention | -0.11 | 0.02 | [-0.15, -0.07] | -4.65 | 56.06 | < 0.001 |
| | Benefactor’s cost×Extra information | -0.02 | 0.01 | [-0.04, 0.00] | -1.40 | 82.20 | 0.164 |
| **Guilt** | Benefactor’s cost | 0.48 | 0.04 | [0.40, 0.56] | 12.80 | 56.00 | < 0.001 |
| | Extra information about benefactor’s intention | -0.23 | 0.03 | [-0.29, -0.17] | -8.66 | 56.00 | < 0.001 |
| | Benefactor’s cost×Extra information | -0.07 | 0.01 | [-0.09, -0.05] | -4.74 | 55.95 | < 0.001 |
| **Obligation** | Benefactor’s cost | 0.30 | 0.04 | [0.22, 0.38] | 7.27 | 56.01 | < 0.001 |
| | Extra information about benefactor’s intention | 0.25 | 0.04 | [0.17, 0.33] | 6.25 | 56.01 | < 0.001 |
| | Benefactor’s cost×Extra information | 0.10 | 0.02 | [0.06, 0.14] | 6.46 | 55.87 | < 0.001 |
| **Reciprocity** | Benefactor’s cost | 0.63 | 0.04 | [0.55, 0.71] | 15.67 | 56.00 | < 0.001 |
| | Extra information about benefactor’s intention | -0.05 | 0.01 | [-0.07, -0.03] | -3.23 | 57.79 | < 0.001 |
| | Benefactor’s cost×Extra information | -0.04 | 0.01 | [-0.06, -0.02] | -2.92 | 82.67 | < 0.001 |
| **Decision to reject help** | Benefactor’s cost | -0.87 | 0.01 | [-0.89, -0.85] | -1262.41 | 1579 | < 0.001 |
| | Extra information about benefactor’s intention | 0.24 | 0.01 | [0.22, 0.26] | 351.12 | 1579 | < 0.001 |
| | Benefactor’s cost×Extra information | 0.02 | 0.01 | [0.00, 0.04] | 21.73 | 1579 | < 0.001 |

Note: Each analysis was carried out using linear mixed model, two-tailed, uncorrected, with independent hypotheses. Each hypothesis testing should have no association with raising the false positive rate of a separate hypothesis.

**Table S6. The effects of the extra information about benefactor's intention, benefactor's cost, and efficiency on participants' emotional and behavioral responses (Study 2b)**

| Dependent variable | Predictors | Beta | SE | 95%CI | *t* (*z*) | *df* | *p* |
|---|---|---|---|---|---|---|---|
| **Second-order belief** | Benefactor's cost | 0.46 | 0.02 | [0.42, 0.50] | 29.91 | 106.90 | <0.001 |
| | Extra information about benefactor's intention | 0.51 | 0.02 | [0.47, 0.55] | 33.12 | 106.31 | <0.001 |
| | Efficiency | 0.02 | 0.02 | [-0.02, 0.06] | 1.54 | 107.02 | 0.124 |
| | Benefactor's cost×Extra information | 0.26 | 0.02 | [0.22, 0.30] | 16.52 | 71.36 | <0.001 |
| | Efficiency×Benefactor's cost | 0.01 | 0.02 | [-0.03, 0.05] | 0.42 | 104.43 | 0.678 |
| | Efficiency× Extra information | 0.01 | 0.02 | [-0.03, 0.05] | 0.89 | 300.91 | 0.376 |
| | Efficiency×Extra information ×Extra information | 0.02 | 0.02 | [-0.02, 0.06] | 1.36 | 255.83 | 0.174 |
| **Perceived care** | Benefactor's cost | 0.73 | 0.01 | [0.71, 0.75] | 55.50 | 106.88 | <0.001 |
| | Extra information about benefactor's intention | -0.25 | 0.01 | [-0.27, -0.23] | -19.37 | 106.52 | <0.001 |
| | Efficiency | 0.04 | 0.01 | [0.02, 0.06] | 3.30 | 85.22 | 0.001 |
| | Benefactor's cost×Extra information | -0.08 | 0.01 | [-0.10, -0.06] | -6.12 | 100.05 | <0.001 |
| | Efficiency×Benefactor's cost | 0.02 | 0.01 | [0.00, 0.04] | 1.71 | 245.86 | 0.087 |
| | Efficiency× Extra information | -0.01 | 0.01 | [-0.03, 0.01] | -0.72 | 84.53 | 0.469 |
| | Efficiency×Extra information ×Extra information | 0.00 | 0.01 | [-0.02, 0.02] | -0.25 | 328.55 | 0.803 |
| **Gratitude** | Benefactor's cost | 0.60 | 0.02 | [0.56, 0.64] | 39.33 | 107.10 | <0.001 |
| | Extra information about benefactor's intention | -0.22 | 0.02 | [-0.26, -0.18] | -14.96 | 106.36 | <0.001 |
| | Efficiency | 0.05 | 0.02 | [0.01, 0.09] | 3.27 | 67.60 | 0.001 |
| | Benefactor's cost×Extra information | -0.05 | 0.02 | [-0.09, -0.01] | -3.54 | 101.84 | <0.001 |
| | Efficiency×Benefactor's cost | 0.00 | 0.02 | [-0.04, 0.04] | 0.01 | 72.71 | 0.989 |
| | Efficiency× Extra information | -0.01 | 0.02 | [-0.05, 0.03] | -0.86 | 72.33 | 0.388 |
| | Efficiency×Extra information ×Extra information | 0.01 | 0.02 | [-0.03, 0.05] | 0.40 | 117.94 | 0.688 |
| **Indebtedness** | Benefactor's cost | 0.60 | 0.01 | [0.58, 0.62] | 40.90 | 106.47 | <0.001 |
| | Extra information about benefactor's intention | -0.11 | 0.01 | [-0.13, -0.09] | -7.88 | 105.87 | <0.001 |
| | Efficiency | 0.04 | 0.01 | [0.02, 0.06] | 2.65 | 67.35 | 0.008 |
| | Benefactor's cost×Extra information | -0.03 | 0.01 | [-0.05, -0.01] | -1.79 | 100.16 | 0.074 |
| | Efficiency×Benefactor's cost | 0.03 | 0.02 | [-0.01, 0.07] | 1.89 | 100.93 | 0.059 |
| | Efficiency× Extra information | -0.02 | 0.01 | [-0.04, 0.00] | -1.25 | 95.37 | 0.210 |
| | Efficiency×Extra information ×Extra information | -0.02 | 0.02 | [-0.06, 0.02] | -1.39 | 123.68 | 0.165 |
| **Guilt** | Benefactor's cost | 0.48 | 0.01 | [0.46, 0.50] | 35.83 | 107.42 | <0.001 |
| | Extra information about benefactor's intention | -0.23 | 0.01 | [-0.25, -0.21] | -17.01 | 106.42 | <0.001 |

| | | | | | | | |
|---|---|---|---|---|---|---|---|
| | Efficiency | 0.01 | 0.01 | [-0.01, 0.03] | 0.74 | 60.12 | 0.458 |
| | Benefactor’s cost×Extra information | -0.07 | 0.01 | [-0.09, -0.05] | -5.17 | 98.70 | <0.001 |
| | Efficiency×Benefactor’s cost | 0.01 | 0.01 | [-0.01, 0.03] | 0.57 | 86.44 | 0.567 |
| | Efficiency× Extra information | -0.02 | 0.01 | [-0.04, 0.00] | -1.32 | 1156 | 0.188 |
| | Efficiency×Extra information ×Extra information | 0.00 | 0.01 | [-0.02, 0.02] | 0.22 | 238.02 | 0.828 |
| **Obligation** | Benefactor's cost | 0.30 | 0.02 | [0.26, 0.34] | 19.60 | 107.03 | <0.001 |
| | Extra information about benefactor’s intention | 0.25 | 0.02 | [0.21, 0.29] | 16.50 | 106.92 | <0.001 |
| | Efficiency | 0.00 | 0.02 | [-0.04, 0.04] | -0.03 | 58.64 | 0.975 |
| | Benefactor’s cost×Extra information | 0.10 | 0.02 | [0.06, 0.14] | 6.43 | 101.83 | <0.001 |
| | Efficiency×Benefactor’s cost | 0.01 | 0.02 | [-0.03, 0.05] | 0.85 | 72.95 | 0.396 |
| | Efficiency× Extra information | -0.01 | 0.02 | [-0.05, 0.03] | -0.72 | 45.49 | 0.470 |
| | Efficiency×Extra information ×Extra information | -0.01 | 0.02 | [-0.05, 0.03] | -0.35 | 103.61 | 0.726 |
| **Reciprocity** | Benefactor's cost | 0.65 | 0.01 | [0.63, 0.67] | 48.73 | 108.92 | <0.001 |
| | Extra information about benefactor’s intention | -0.05 | 0.01 | [-0.07, -0.03] | -4.00 | 107.10 | <0.001 |
| | Efficiency | 0.08 | 0.01 | [0.06, 0.10] | 5.86 | 59.09 | <0.001 |
| | Benefactor’s cost×Extra information | -0.04 | 0.01 | [-0.06, -0.02] | -2.74 | 129.93 | 0.006 |
| | Efficiency×Benefactor’s cost | 0.02 | 0.01 | [0.00, 0.04] | 1.69 | 86.04 | 0.090 |
| | Efficiency× Extra information | 0.00 | 0.01 | [-0.02, 0.02] | -0.08 | 30.32 | 0.940 |
| | Efficiency×Extra information ×Extra information | 0.01 | 0.01 | [-0.01, 0.03] | 0.63 | 208.20 | 0.528 |
| **Decision to reject help** | Benefactor's cost | -0.70 | 0.07 | [-0.84, -0.56] | -9.61 | 2758 | <0.001 |
| | Extra information about benefactor’s intention | 0.27 | 0.07 | [0.13, 0.41] | 3.92 | 2758 | <0.001 |
| | Efficiency | -0.46 | 0.07 | [-0.60, -0.32] | -6.49 | 2758 | <0.001 |
| | Benefactor’s cost×Extra information | 0.01 | 0.07 | [-0.13, 0.15] | 0.13 | 2758 | 0.895 |
| | Efficiency×Benefactor’s cost | -0.11 | 0.07 | [-0.25, 0.03] | -1.51 | 2758 | 0.131 |
| | Efficiency× Extra information | 0.05 | 0.07 | [-0.09, 0.19] | 0.67 | 2758 | 0.500 |
| | Efficiency×Extra information ×Extra information | -0.04 | 0.07 | [-0.18, 0.10] | -0.53 | 2758 | 0.594 |

Note: Each analysis was carried out using linear mixed model, two-tailed, uncorrected, with independent hypotheses. Each hypothesis testing should have no association with raising the false positive rate of a separate hypothesis. In the models of this table, we only included random intercepts for participants due to convergence issues with models that additionally included random slopes.

**Table S7. The effects of the extra information about benefactor's intention and benefactor's cost on participants' emotional and behavioral responses (fMRI study)**

| Dependent variable | Predictors | Beta | SE | 95%CI | *t* (*z*) | *df* | *p* |
|---|---|---|---|---|---|---|---|
| **Second-order belief** | Benefactor's cost | 0.44 | 0.02 | [0.40, 0.48] | 22.20 | 52.14 | < 0.001 |
| | Extra information about benefactor’s intention | 0.65 | 0.04 | [0.57, 0.73] | 15.88 | 52.00 | < 0.001 |
| | Benefactor’s cost×Extra information | 0.29 | 0.02 | [0.25, 0.33] | 15.21 | 52.13 | < 0.001 |
| **Perceived care** | Benefactor's cost | 0.66 | 0.03 | [0.60, 0.72] | 19.23 | 51.80 | < 0.001 |
| | Extra information about benefactor’s intention | -0.29 | 0.03 | [-0.35, -0.23] | -10.00 | 52.07 | < 0.001 |
| | Benefactor’s cost×Extra information | -0.09 | 0.02 | [-0.13, -0.05] | -4.16 | 52.22 | < 0.001 |
| **Gratitude** | Benefactor's cost | 0.66 | 0.03 | [0.60, 0.72] | 20.45 | 51.15 | < 0.001 |
| | Extra information about benefactor’s intention | -0.28 | 0.03 | [-0.34, -0.22] | -9.61 | 51.95 | < 0.001 |
| | Benefactor’s cost×Extra information | -0.06 | 0.02 | [-0.10, -0.02] | -2.77 | 52.19 | 0.008 |
| **Indebtedness** | Benefactor’s cost | 0.56 | 0.04 | [0.48, 0.64] | 15.15 | 52.10 | < 0.001 |
| | Extra information about benefactor’s intention | -0.11 | 0.03 | [-0.17, -0.05] | -3.45 | 52.18 | 0.001 |
| | Benefactor’s cost×Extra information | 0.02 | 0.02 | [-0.02, 0.06] | 0.87 | 66.52 | 0.388 |
| **Guilt** | Benefactor's cost | 0.46 | 0.04 | [0.38, 0.54] | 11.89 | 52.01 | < 0.001 |
| | Extra information about benefactor’s intention | -0.26 | 0.04 | [-0.34, -0.18] | -6.64 | 52.03 | < 0.001 |
| | Benefactor’s cost×Extra information | -0.09 | 0.02 | [-0.13, -0.05] | -4.05 | 52.03 | < 0.001 |
| **Obligation** | Benefactor’s cost | 0.23 | 0.05 | [0.13, 0.33] | 4.89 | 52.06 | < 0.001 |
| | Extra information about benefactor’s intention | 0.29 | 0.04 | [0.21, 0.37] | 6.67 | 51.99 | < 0.001 |
| | Benefactor’s cost×Extra information | 0.17 | 0.02 | [0.13, 0.21] | 7.53 | 58.65 | < 0.001 |
| **Reciprocity** | Benefactor’s cost | 0.77 | 0.03 | [0.71, 0.83] | 23.56 | 52.04 | < 0.001 |
| | Extra information about benefactor’s intention | -0.07 | 0.02 | [-0.11, -0.03] | -4.21 | 52.09 | < 0.001 |
| | Benefactor’s cost×Extra information | -0.02 | 0.01 | [-0.04, 0.00] | -2.65 | 100.39 | 0.009 |

Note: Each analysis was carried out using linear mixed model, two-tailed, uncorrected, with independent hypotheses. Each hypothesis testing should have no association with raising the false positive rate of a separate hypothesis.

**Table S8. Correlations between appraisals and emotions (within participant)**

| | | Second-order belief | Obligation | Indebtedness | Guilt | Gratitude | Perceived care |
|---|---|---|---|---|---|---|---|
| Second-order belief | average *r* | - | 0.47 | 0.28 | 0.09 | 0.13 | 0.13 |
| | 95%CI | | [0.41,0.54] | [0.20,0.35] | [0.01,0.17] | [0.05,0.21] | [0.05,0.21] |
| | $t(107)$ | - | 12.32 | 6.79 | 2.32 | 3.41 | 3.26 |
| | $p_{FDR}$ | - | < 0.001 | < 0.001 | 0.028 | < 0.001 | 0.001 |
| Obligation | average *r* | - | - | 0.27 | 0.10 | 0.04 | 0.05 |
| | 95%CI | | | [0.18,0.36] | [-0.01,0.21] | [-0.05,0.14] | [-0.06,0.16] |
| | $t(107)$ | - | - | 5.27 | 2.17 | 1.10 | 1.36 |
| | $p_{FDR}$ | - | - | < 0.001 | 0.037 | 0.275 | 0.188 |
| Indebtedness | average *r* | - | - | - | 0.66 | 0.55 | 0.67 |
| | 95%CI | | | | [0.60,0.72] | [0.48,0.62] | [0.59,0.74] |
| | $t(107)$ | - | - | - | 17.63 | 12.82 | 15.50 |
| | $p_{FDR}$ | - | - | - | < 0.001 | < 0.001 | < 0.001 |
| Guilt | average *r* | - | - | - | - | 0.64 | 0.75 |
| | 95%CI | | | | | [0.58,0.70] | [0.69,0.80] |
| | $t(107)$ | - | - | - | - | 16.49 | 20.13 |
| | $p_{FDR}$ | - | - | - | - | < 0.001 | < 0.001 |
| Gratitude | average *r* | - | - | - | - | - | 0.80 |
| | 95%CI | | | | | | [0.77,0.84] |
| | $t(107)$ | - | - | -- | - | - | 25.54 |
| | $p_{FDR}$ | - | - | - | - | - | < 0.001 |
| Perceived care | average *r* | - | - | - | - | - | - |
| | 95%CI | | | | | | |
| | $t(107)$ | - | - | - | - | - | - |
| | $p_{FDR}$ | - | - | - | - | - | - |

Note: For within-participant analysis, for each pair of these six variables, we estimated the Pearson correlation for each participant, transformed the data using a fisher *r* to *z* transformation, and then conducted a one-sample *t*-test using *z* values of all participants to evaluate whether the two variables were significantly correlated at the group level. Two-tailed, FDR corrected.

**Table S9. Correlations between appraisals and emotions (between participant)**

| | | Second-order belief | Obligation | Indebtedness | Guilt | Gratitude | Perceived care |
|---|---|---|---|---|---|---|---|
| Second-order belief | *r* | - | 0.36 | 0.25 | 0.27 | 0.00 | 0.17 |
| | 95%CI | | [0.18,0.51] | [0.06,0.42] | [0.09,0.44] | [-0.19,0.19] | [-0.02,0.34] |
| | *t*(106) | - | 3.96 | 2.64 | 2.89 | 0.00 | 1.73 |
| | $p_{FDR}$ | - | < 0.001 | 0.012 | 0.075 | 0.996 | 0.108 |
| Obligation | *r* | - | - | 0.44 | 0.42 | -0.06 | 0.03 |
| | 95%CI | | | [0.27,0.58] | [0.25,0.56] | [-0.24,0.14] | [-0.16,0.21] |
| | *t*(106) | - | - | 4.99 | 4.74 | -0.57 | 0.32 |
| | $p_{FDR}$ | - | - | < 0.001 | < 0.001 | 0.657 | 0.805 |
| Indebtedness | *r* | - | - | - | 0.81 | 0.41 | 0.53 |
| | 95%CI | | | | [0.73,0.87] | [0.24,0.56] | [0.37,0.65] |
| | *t*(106) | - | - | - | 14.28 | 4.69 | 6.36 |
| | $p_{FDR}$ | - | - | - | < 0.001 | < 0.001 | < 0.001 |
| Guilt | *r* | - | - | - | - | 0.40 | 0.41 |
| | 95%CI | | | | | [0.23,0.55] | [0.24,0.56] |
| | *t*(106) | - | - | - | -- | 4.52 | 4.62 |
| | $p_{FDR}$ | - | - | - | - | < 0.001 | < 0.001 |
| Gratitude | *r* | - | - | - | - | - | 0.68 |
| | 95%CI | | | | | | [0.56,0.77] |
| | *t*(106) | - | - | - | - | - | 9.58 |
| | $p_{FDR}$ | - | - | - | - | - | < 0.001 |
| Perceived care | *r* | - | - | - | - | - | - |
| | *t*(106) | - | - | - | -- | - | - |
| | $p_{FDR}$ | - | - | - | - | - | - |

Note: For between-participant analysis, for each of the six variables, we computed the average value of the variable across all trials for each participant. We then estimated the Pearson correlations between each pair of variables based on variability across participants. Two-tailed, FDR corrected.

**Table S10. Model comparison for reciprocity decisions**

| Model | Model description | Average Sum of Squared Error | | |
|---|---|---|---|---|
| | | Study 2a | Study 2b | Combined |
| **Model 1.1** | Nonlinear version | 4331.00 | 3938.60 | 4123.90 |
| Model 1.2 | Linear version | 5550.93 | 7177.44 | 6409.36 |
| Model 1.3 | Only communal concern | 4773.74 | 5085.24 | 4938.14 |
| Model 1.4 | Only obligation | 40365.60 | 33791.45 | 36895.91 |
| Model 1.5 | Three separate parameters independently weighted communal concern and obligation | 4521.81 | 4134.60 | 4317.45 |
| Model 1.6 | Three separate parameters independently weighted greedy, communal concern and obligation | 4466.52 | 3840.01 | 4135.86 |
| Model 1.7 | Reciprocity according to benefactor's cost | 5475.37 | 5003.66 | 5226.41 |
| Model 1.8 | Inequity aversion model | 13726.42 | 10080.51 | 11802.19 |

**Table S11. Model comparison for reciprocity decisions**

| Model | Model description | Average AIC | | | Wilcoxon rank sum test | | |
|---|---|---|---|---|---|---|---|
| | | Study 2a | Study 2b | Combined | Wilcoxon W | Effect size | $p_{FDR}$ |
| **Model 1.1** | Nonlinear version | 117.51 | 130.49 | 124.36 | - | - | - |
| Model 1.2 | Linear version | 125.22 | 148.06 | 137.27 | 1108 | 0.62 | < 0.001 |
| Model 1.3 | Only communal concern | 117.16 | 136.07 | 127.14 | 2170 | 0.26 | 0.018 |
| Model 1.4 | Only obligation | 175.24 | 190.87 | 183.49 | 10 | 1.00 | < 0.001 |
| Model 1.5 | Three separate parameters independently weighted communal concern and obligation | 121.34 | 135.09 | 128.59 | 1148 | 0.61 | < 0.001 |
| Model 1.6 | Three separate parameters independently weighted greedy, communal concern and obligation | 121.23 | 132.08 | 126.96 | 832 | 0.72 | < 0.001 |
| Model 1.7 | Reciprocity according to benefactor's cost | 124.01 | 133.75 | 129.15 | 853 | 0.71 | < 0.001 |
| Model 1.8 | Inequity aversion model | 150.92 | 160.58 | 156.02 | 135 | 0.95 | < 0.001 |

Note: The average AIC value of each model was compared with that of Model 1.1 using Wilcoxon rank sum test, two-tailed, FDR corrected.

**Table S12. Model comparison for help-acceptance decisions**

| | | Log Likelihood | | |
|---|---|---|---|---|
| Model | Model description | Study 2a | Study 2b | Combined |
| **Model 2.1** | Full model | -206.97 | -293.05 | -251.24 |
| Model 2.2 | Only communal concern | -202.92 | -303.73 | -254.77 |
| Model 2.3 | Only obligation | -237.20 | -339.40 | -289.76 |
| Model 2.4 | Three separate parameters independently weighted communal concern and obligation | -206.97 | -297.05 | -253.30 |
| Model 2.5 | Three separate parameters independently weighted greedy, communal concern and obligation | -208.69 | -293.17 | -253.14 |

**Table S13. Model comparison for help-acceptance decisions**

| | | Average AIC | | | Wilcoxon rank sum test | | |
|---|---|---|---|---|---|---|---|
| Model | Model description | Study 2a | Study 2b | Combined | Wilcoxon W | Effect size | $p_{FDR}$ |
| **Model 2.1** | Full model | 555.95 | 760.09 | 660.93 | - | - | - |
| Model 2.2 | Only communal concern | 549.85 | 775.47 | 665.88 | 4696 | 0.06 | 0.203 |
| Model 2.3 | Only obligation | 570.40 | 790.80 | 683.75 | 3950 | 0.10 | 0.089 |
| Model 2.4 | Three separate parameters independently weighted communal concern and obligation | 605.95 | 818.10 | 715.06 | 2065 | 0.65 | < 0.001 |
| Model 2.5 | Three separate parameters independently weighted greedy, communal concern and obligation | 607.56 | 821.33 | 717.50 | 2012 | 0.67 | < 0.001 |

Note: The average AIC value of each model was compared with that of Model 1.1 using Wilcoxon rank sum test, two-tailed, FDR corrected.

**Table S14. Parameter recovery for reciprocity decisions**

| Model | Model description | Study 2a | | | Study 2b | | | Total | | |
|---|---|---|---|---|---|---|---|---|---|---|
| | | $r \pm SE$ | 95%CI | $p_{FDR}$ | $r \pm SE$ | 95%CI | $p_{FDR}$ | $r \pm SE$ | 95%CI | $p_{FDR}$ |
| **Model 1.1** | Nonlinear version | .93±.07 | [0.79, 1.07] | <0.001 | .94±.08 | [0.78, 1.10] | <0.001 | .94±.07 | [0.80, 1.08] | <0.001 |
| Model 1.2 | Linear version | .29±.20 | [-0.10, 0.68] | <0.001 | .36±.20 | [-0.03, 0.75] | <0.001 | .33±.20 | [-0.06, 0.72] | <0.001 |
| Model 1.3 | Only Communal Concern | .99±.02 | [0.95, 1.03] | <0.001 | .90±.09 | [0.72, 1.08] | <0.001 | .93±.06 | [0.81, 1.05] | <0.001 |
| Model 1.4 | Only Obligation | .99±.02 | [0.95, 1.03] | <0.001 | .98±.04 | [0.90, 1.06] | <0.001 | .99±.03 | [0.93, 1.05] | <0.001 |
| Model 1.5 | Three separate parameters independently weighted communal concern and obligation | .56±.28 | [0.01, 1.11] | <0.001 | .65±.25 | [0.16, 1.14] | <0.001 | .61±.27 | [0.08, 1.14] | <0.001 |
| Model 1.6 | Three separate parameters independently weighted greedy, communal concern and obligation | .52±.28 | [-0.03, 1.07] | <0.001 | .70±.23 | [0.25, 1.15] | <0.001 | .57±.27 | [0.04, 1.10] | <0.001 |
| Model 1.7 | Reciprocity according to benefactor's cost | .80±.21 | [0.39, 1.21] | <0.001 | .82±.19 | [0.45, 1.19] | <0.001 | .81±.20 | [0.42, 1.20] | <0.001 |
| Model 1.8 | Inequity aversion model | .76±.22 | [0.33, 1.19] | <0.001 | .73±.24 | [0.26, 1.20] | <0.001 | .74±.23 | [0.29, 1.19] | <0.001 |

Note: Pearson correlation analysis, two-tailed, FDR corrected.

**Table S15. Parameter recovery for help-acceptance decisions**

| Model | Model description | Study 2a | | | Study 2b | | | Total | | |
|---|---|---|---|---|---|---|---|---|---|---|
| | | $r \pm SE$ | 95%CI | $p_{FDR}$ | $r \pm SE$ | 95%CI | $p_{FDR}$ | $r \pm SE$ | 95%CI | $p_{FDR}$ |
| **Model 2.1** | Full model | .45±.37 | [-0.28, 1.18] | <0.001 | .41±.38 | [-0.33, 1.15] | <0.001 | .43±.40 | [-0.35, 1.21] | <0.001 |
| Model 2.2 | Only Communal Concern | .73±.43 | [-0.11, 1.57] | <0.001 | .50±.48 | [-0.44, 1.44] | <0.001 | .62±.46 | [-0.28, 1.52] | <0.001 |
| Model 2.3 | Only Obligation | -.05±6.94 | [-13.65, 13.55] | 0.647 | .29±.67 | [-1.02, 1.60] | 0.002 | -.02±4.80 | [-9.43, 9.39] | 0.738 |
| Model 2.4 | Three separate parameters independently weighted communal concern and obligation | .64±.32 | [0.01, 1.27] | <0.001 | .62±.28 | [0.07, 1.17] | <0.001 | .63±.30 | [0.04, 1.22] | <0.001 |
| Model 2.5 | Three separate parameters independently weighted greedy, communal concern and obligation | .46±.32 | [-0.17, 1.09] | <0.001 | .43±.35 | [-0.26, 1.12] | <0.001 | .45±.34 | [-0.22, 1.12] | <0.001 |

Note: Pearson correlation analysis, two-tailed, FDR corrected.

**Table S16. Model estimated parameters for reciprocity decisions**

| | Study 2a | | Study 2b | | Combined | |
|---|---|---|---|---|---|---|
| Parameters | Mean | SE | Mean | SE | Mean | SE |
| $\theta$ | 0.06 | 0.01 | 0.10 | 0.02 | 0.08 | 0.01 |
| $W_{Communal}$ ($\phi$) | 0.83 | 0.03 | 0.75 | 0.02 | 0.79 | 0.02 |
| $W_{Obligation}$ ($1 - \phi$) | 0.17 | 0.03 | 0.25 | 0.02 | 0.21 | 0.02 |
| $\kappa$ | 0.21 | 0.03 | 0.41 | 0.04 | 0.32 | 0.01 |

**Table S17. Model estimated parameters for help-acceptance decisions**

| | Study 2a | | Study 2b | | Combined | |
|---|---|---|---|---|---|---|
| Parameters | Mean | SE | Mean | SE | Mean | SE |
| $\theta$ | 0.39 | 0.03 | 0.37 | 0.04 | 0.37 | 0.02 |
| $W_{Communal}$ ($\phi$) | -0.16 | 0.08 | 0.17 | 0.09 | 0.01 | 0.06 |
| $W_{Obligation}$ ($1 - \|\phi\|$) | 0.55 | 0.05 | 0.35 | 0.04 | 0.44 | 0.03 |
| $\kappa$ | 0.46 | 0.03 | 0.43 | 0.04 | 0.45 | 0.02 |

**Table S18. Results of whole-brain analysis of fMRI data**

| Regions | Hemisphere | $t$ | $p_{FWE}$ | Cluster size (voxels) | MNI coordinates | | |
|---|---|---|---|---|---|---|---|
| | | | | | x | y | z |
| **Regions responded parametrically to the amount of reciprocity** | | | | | | | |
| Left dlPFC | L | 5.93 | < 0.001 | 209 | -45 | 5 | 29 |
| Right dlPFC | R | 4.91 | 0.004 | 138 | 45 | 11 | 35 |
| | | | | | 39 | 8 | 38 |
| | | | | | 45 | 35 | 11 |
| Left IPL | L | 4.25 | 0.046 | 48 | -54 | -40 | 53 |
| Right IPL | R | 4.80 | 0.005 | 130 | 51 | -28 | 47 |
| Precuneus-MOG-ITG | R | 6.59 | < 0.001 | 958 | 51 | -52 | -13 |
| MOG | L | 5.34 | < 0.001 | 637 | -30 | -67 | 29 |
| ITG | L | 5.30 | < 0.001 | 479 | -45 | -61 | -13 |
| Cerebellum | L | 3.99 | 0.048 | 38 | -27 | -61 | -34 |
| Cerebellum | R | 4.55 | 0.043 | 45 | 6 | -31 | -22 |
| **Regions responded parametrically to communal concern ($\omega_B$)** | | | | | | | |
| vmPFC | - | 4.19 | 0.047 | 41 | 0 | 35 | -22 |
| aINS | L | 4.60 | 0.021 | 98 | -24 | 11 | -19 |
| Left dlPFC | L | 4.65 | 0.002 | 161 | -48 | 20 | 26 |
| | | | | | -24 | 29 | 56 |
| Right dlPFC | R | 5.24 | < 0.001 | 251 | 45 | 11 | 38 |
| Precuneus | R | 4.46 | < 0.001 | 683 | 3 | -46 | 38 |
| ITG | R | 4.85 | 0.006 | 128 | 48 | -46 | -16 |
| ITG | L | 4.64 | < 0.001 | 198 | -54 | -76 | -7 |
| MOG | L | 4.89 | < 0.001 | 490 | -30 | -76 | 32 |
| Calcarine | R | 4.29 | 0.045 | 61 | 9 | -91 | 5 |
| **Regions identified in parametric contrast for obligation ($E_B''$)** | | | | | | | |
| dmPFC | L | 4.39 | 0.044 | 31 | -9 | 44 | 41 |
| Left TPJ | L | 3.86 | 0.049 | 42 | -57 | -61 | 26 |

Note: dlPFC = dorsolateral prefrontal cortex; IPL = inferior parietal lobule; MOG = middle occipital gyrus; ITG = inferior temporal gyrus; vmPFC = ventromedial prefrontal cortex; aINS = anterior insula; dmPFC = dorsomedial prefrontal lobe; TPJ = temporal parietal junction. One-sample $t$-tests at second-level analysis, two-tailed. All brain maps were thresholded using cluster correction FWE $p < 0.05$ with a cluster-forming threshold of $p < 0.001$.

**Table S19. The meaning of symbols for variables in the computational model**

| Variable | Meaning |
|---|---|
| $D$ | Decision over choice space, e.g., the benefactor's cost, the beneficiary's amount of reciprocity and the beneficiary's decision of accepting help |
| $\theta$ | Greed sensitivity |
| $\phi$ | Mixture weight of $U_{Communal}$ and $U_{Obligation}$ |
| $\gamma$ | Endowment size |
| $\pi$ | Self interest |
| $\mu$ | The efficiency of help |
| $E''$ | Second-order belief of how much the benefactor expects |
| $\omega$ | Perceived care |
| $\kappa$ | The influence of second-order belief ($E''$) on perceived care ($\omega$). Higher indicates lower perceived care. |
| $\lambda$ | Inverse temperature parameter |
| n | Total number of trials |
| $t$ | Trial number |

**Table S20. Classifications for words in the definition of indebtedness**

| Classification | Word | English word | Weight | Frequency to be Classified in each level (%) | | | | |
|---|---|---|---|---|---|---|---|---|
| | | | | Appraisal | Emotion | Behavior | Person | Other |
| Appraisal | 损失 | Loss | 0.075 | 45.0 | 7.5 | 36.3 | 0.0 | 11.3 |
| | 代价 | Cost | 0.033 | 41.3 | 5.0 | 17.5 | 2.5 | 33.8 |
| | 不好 | Bad | 0.014 | 55.0 | 30.0 | 0.0 | 1.3 | 13.8 |
| | 受损 | Harm | 0.012 | 45.0 | 6.3 | 45.0 | 0.0 | 3.8 |
| | 很大 | Great | 0.012 | 51.3 | 5.0 | 2.5 | 2.5 | 38.8 |
| | 不必要 | Unnecessary | 0.010 | 46.3 | 12.5 | 5.0 | 1.3 | 35.0 |
| Emotion | 愧疚 | Guilt | 0.269 | 1.3 | 97.5 | 0.0 | 0.0 | 1.3 |
| | 内疚 | Guilt | 0.192 | 0.0 | 98.8 | 0.0 | 1.3 | 0.0 |
| | 亏欠 | Feel indebted | 0.154 | 25.0 | 46.3 | 26.3 | 0.0 | 2.5 |
| | 感觉 | Feel | 0.120 | 10.0 | 66.3 | 15.0 | 0.0 | 8.8 |
| | 感到 | Feel | 0.102 | 5.0 | 66.3 | 20.0 | 0.0 | 8.8 |
| | 觉得 | Feel | 0.074 | 15.0 | 53.8 | 17.5 | 0.0 | 13.8 |
| | 对不起 | Feel sorry | 0.068 | 8.8 | 62.5 | 15.0 | 0.0 | 13.8 |
| | 想要 | Want to | 0.052 | 6.3 | 56.3 | 32.5 | 1.3 | 3.8 |
| | 不安 | Uneasy | 0.047 | 0.0 | 97.5 | 1.3 | 0.0 | 1.3 |
| | 麻烦 | Trouble | 0.041 | 26.3 | 36.3 | 21.3 | 2.5 | 13.8 |
| | 难受 | Uncomfortable | 0.040 | 0.0 | 98.8 | 0.0 | 1.3 | 0.0 |
| | 负罪感 | Guilt | 0.034 | 3.8 | 93.8 | 0.0 | 1.3 | 1.3 |
| | 自责 | Guilt | 0.033 | 5.0 | 85.0 | 8.8 | 1.3 | 0.0 |
| | 过意不去 | Feel sorry | 0.026 | 2.5 | 95.0 | 0.0 | 1.3 | 1.3 |
| | 有愧 | Guilt | 0.023 | 1.3 | 95.0 | 2.5 | 0.0 | 1.3 |
| | 感激 | Gratitude | 0.022 | 1.3 | 86.3 | 12.5 | 0.0 | 0.0 |
| | 不好意思 | Feel sorry | 0.020 | 1.3 | 91.3 | 2.5 | 3.8 | 1.3 |
| | 抱歉 | Feel sorry | 0.017 | 2.5 | 87.5 | 8.8 | 1.3 | 0.0 |
| | 不舒服 | Uncomfortable | 0.016 | 5.0 | 92.5 | 0.0 | 1.3 | 1.3 |
| | 心里 | In the heart | 0.013 | 3.8 | 41.3 | 25.0 | 3.8 | 26.3 |
| | 压力 | Pressure | 0.013 | 10.0 | 72.5 | 3.8 | 1.3 | 12.5 |
| | 情感 | Emotion | 0.013 | 7.5 | 70.0 | 0.0 | 0.0 | 22.5 |
| | 内疚感 | Guilt | 0.013 | 1.3 | 96.3 | 0.0 | 2.5 | 0.0 |
| | 负担 | Burden | 0.012 | 16.3 | 37.5 | 33.8 | 0.0 | 12.5 |
| | 痛苦 | Painful | 0.011 | 1.3 | 95.0 | 3.8 | 0.0 | 0.0 |
| | 强烈 | Strong | 0.010 | 20.0 | 57.5 | 2.5 | 1.3 | 18.8 |
| | 希望 | Want to | 0.010 | 16.3 | 56.3 | 17.5 | 0.0 | 10.0 |
| | 歉疚 | Guilt | 0.010 | 1.3 | 96.3 | 1.3 | 1.3 | 0.0 |
| Behavior | 帮助 | Help | 0.312 | 3.8 | 0.0 | 93.8 | 2.5 | 0.0 |
| | 伤害 | Harm | 0.101 | 15.0 | 11.3 | 73.8 | 0.0 | 0.0 |
| | 付出 | Cost | 0.091 | 10.0 | 3.8 | 82.5 | 1.3 | 2.5 |
| | 负债 | Be in debt | 0.090 | 22.5 | 13.8 | 46.3 | 2.5 | 15.0 |
| | 回报 | Repay | 0.068 | 20.0 | 3.8 | 66.3 | 1.3 | 8.8 |

| Classification | Word | English word | Weight | Frequency to be Classified in each level (%) | | | | |
|---|---|---|---|---|---|---|---|---|
| | | | | Appraisal | Emotion | Behavior | Person | Other |
| | 造成 | Cause | 0.067 | 15.0 | 0.0 | 78.8 | 0.0 | 6.3 |
| | 损害 | Harm | 0.065 | 22.5 | 5.0 | 68.8 | 0.0 | 3.8 |
| | 受到 | Receive | 0.046 | 7.5 | 17.5 | 45.0 | 0.0 | 30.0 |
| | 接受 | Receive | 0.044 | 3.8 | 10.0 | 82.5 | 1.3 | 2.5 |
| | 产生 | Generate | 0.037 | 7.5 | 3.8 | 57.5 | 1.3 | 30.0 |
| | 补偿 | Compensate | 0.037 | 12.5 | 3.8 | 83.8 | 0.0 | 0.0 |
| | 牺牲 | Sacrifice | 0.037 | 15.0 | 2.5 | 75.0 | 1.3 | 6.3 |
| | 偿还 | Repay | 0.034 | 16.3 | 2.5 | 77.5 | 1.3 | 2.5 |
| | 回馈 | Repay | 0.022 | 13.8 | 1.3 | 78.8 | 2.5 | 3.8 |
| | 带来 | Bring | 0.019 | 6.3 | 3.8 | 77.5 | 1.3 | 11.3 |
| | 收到 | Receive | 0.017 | 2.5 | 2.5 | 88.8 | 1.3 | 5.0 |
| | 需要 | Need | 0.017 | 21.3 | 17.5 | 32.5 | 1.3 | 27.5 |
| | 影响 | Influence | 0.016 | 26.3 | 11.3 | 51.3 | 0.0 | 11.3 |
| | 弥补 | Compensate | 0.016 | 5.0 | 10.0 | 83.8 | 1.3 | 0.0 |
| | 行为 | Behavior | 0.015 | 7.5 | 1.3 | 68.8 | 1.3 | 21.3 |
| | 给予 | Give | 0.013 | 3.8 | 0.0 | 93.8 | 0.0 | 2.5 |
| | 报答 | Repay | 0.012 | 8.8 | 7.5 | 81.3 | 1.3 | 1.3 |
| | 得到 | Receive | 0.012 | 6.3 | 2.5 | 82.5 | 1.3 | 7.5 |
| | 付出代价 | Pay the price | 0.012 | 17.5 | 2.5 | 70.0 | 1.3 | 8.8 |
| | 我会 | I will | 0.012 | 16.3 | 7.5 | 47.5 | 5.0 | 23.8 |
| | 做错 | Wrongdoings | 0.012 | 28.8 | 6.3 | 57.5 | 2.5 | 5.0 |
| | 做错事 | Wrongdoings | 0.011 | 17.5 | 5.0 | 72.5 | 0.0 | 5.0 |
| | 失去 | Loss | 0.011 | 15.0 | 10.0 | 67.5 | 0.0 | 7.5 |
| | 导致 | Lead to | 0.011 | 18.8 | 2.5 | 57.5 | 1.3 | 20.0 |
| Person | 别人 | Other | 0.329 | 1.3 | 0.0 | 1.3 | 96.3 | 1.3 |
| | 他人 | Other | 0.218 | 0.0 | 0.0 | 2.5 | 97.5 | 0.0 |
| | 自己 | Self | 0.202 | 0.0 | 0.0 | 0.0 | 98.8 | 1.3 |
| | 对方 | Other | 0.142 | 2.5 | 0.0 | 0.0 | 96.3 | 1.3 |
| | 帮助者 | Benefactor | 0.036 | 2.5 | 1.3 | 2.5 | 88.8 | 5.0 |
| | 其他人 | Other | 0.026 | 1.3 | 0.0 | 2.5 | 95.0 | 1.3 |
| | 自身 | Self | 0.016 | 2.5 | 3.8 | 0.0 | 83.8 | 10.0 |
| Other | 利益 | Benefit | 0.131 | 36.3 | 7.5 | 6.3 | 2.5 | 47.5 |
| | 内心 | In the heart | 0.114 | 3.8 | 43.8 | 0.0 | 2.5 | 50.0 |
| | 因为 | Because | 0.077 | 18.8 | 3.8 | 5.0 | 1.3 | 71.3 |
| | 心存 | In the heart | 0.057 | 6.3 | 36.3 | 2.5 | 1.3 | 53.8 |
| | 人情 | Favor | 0.040 | 17.5 | 20.0 | 7.5 | 2.5 | 52.5 |
| | 东西 | Things | 0.029 | 7.5 | 1.3 | 2.5 | 8.8 | 80.0 |
| | 心理 | Psychological | 0.029 | 7.5 | 38.8 | 1.3 | 2.5 | 50.0 |
| | 事情 | Things | 0.028 | 5.0 | 0.0 | 8.8 | 5.0 | 81.3 |
| | 或者 | Or | 0.024 | 7.5 | 0.0 | 1.3 | 1.3 | 90.0 |

| Classification | Word | English word | Weight | Frequency to be Classified in each level (%) | | | | |
|---|---|---|---|---|---|---|---|---|
| | | | | Appraisal | Emotion | Behavior | Person | Other |
| | 为了 | In order to | 0.023 | 18.8 | 2.5 | 21.3 | 1.3 | 56.3 |
| | 一种 | A kind of | 0.021 | 15.0 | 0.0 | 2.5 | 2.5 | 80.0 |
| | 没有 | No | 0.020 | 25.0 | 0.0 | 2.5 | 1.3 | 71.3 |
| | 某件事 | Something | 0.019 | 3.8 | 0.0 | 6.3 | 15.0 | 75.0 |
| | 有所 | Somewhat | 0.017 | 16.3 | 8.8 | 7.5 | 0.0 | 67.5 |
| | 对于 | For | 0.016 | 21.3 | 2.5 | 10.0 | 2.5 | 63.8 |
| | 一些 | Some | 0.015 | 11.3 | 0.0 | 3.8 | 6.3 | 78.8 |
| | 什么 | What | 0.015 | 7.5 | 2.5 | 1.3 | 2.5 | 86.3 |
| | 应该 | Should | 0.015 | 30.0 | 16.3 | 10.0 | 1.3 | 42.5 |
| | 程度 | Extent | 0.014 | 33.8 | 2.5 | 6.3 | 5.0 | 52.5 |
| | 由于 | Because | 0.013 | 23.8 | 1.3 | 5.0 | 1.3 | 68.8 |
| | 原因 | Reason | 0.013 | 25.0 | 3.8 | 3.8 | 0.0 | 67.5 |
| | 某事 | Something | 0.012 | 5.0 | 0.0 | 5.0 | 11.3 | 78.8 |
| | 某些 | Some | 0.012 | 7.5 | 0.0 | 5.0 | 7.5 | 80.0 |
| | 一定 | Certainly | 0.012 | 27.5 | 7.5 | 7.5 | 2.5 | 55.0 |
| | 是否 | Whether | 0.012 | 37.5 | 1.3 | 2.5 | 1.3 | 57.5 |
| | 感是 | Feel | 0.012 | 10.0 | 28.8 | 3.8 | 0.0 | 57.5 |
| | 心中 | In the heart | 0.011 | 3.8 | 40.0 | 2.5 | 3.8 | 50.0 |
| | 无法 | Cannot | 0.011 | 25.0 | 11.3 | 6.3 | 0.0 | 57.5 |
| | 道德 | Moral | 0.011 | 25.0 | 16.3 | 11.3 | 2.5 | 45.0 |
| | 责任 | Responsibility | 0.010 | 25.0 | 23.8 | 15.0 | 3.8 | 32.5 |
