# Supplementary material for: The psychological, computational, and neural foundations of indebtedness
Source: Nat Commun. 2024 Jan 2;15:68. doi: 10.1038/s41467-023-44286-9 (PMC10762097; doi:10.1038/s41467-023-44286-9)
Supplement: Supplementary file 3 — Reporting Summary [file 41467_2023_44286_MOESM3_ESM.pdf]

nature portfolio

Corresponding author(s): Xiaoxue Gao, Xiaolin Zhou and Luke J. Chang

Last updated by author(s): Sep 21, 2023

Reporting Summary

Nature Portfolio wishes to improve the reproducibility of the work that we publish. This form provides structure for consistency and transparency in reporting. For further information on Nature Portfolio policies, see our Editorial Policies and the Editorial Policy Checklist.

Statistics

For all statistical analyses, confirm that the following items are present in the figure legend, table legend, main text, or Methods section.

n/a	Confirmed	
☐	☒	The exact sample size (*n*) for each experimental group/condition, given as a discrete number and unit of measurement
☐	☒	A statement on whether measurements were taken from distinct samples or whether the same sample was measured repeatedly
☐	☒	The statistical test(s) used AND whether they are one- or two-sided<br>*Only common tests should be described solely by name; describe more complex techniques in the Methods section.*
☐	☒	A description of all covariates tested
☐	☒	A description of any assumptions or corrections, such as tests of normality and adjustment for multiple comparisons
☐	☒	A full description of the statistical parameters including central tendency (e.g. means) or other basic estimates (e.g. regression coefficient) AND variation (e.g. standard deviation) or associated estimates of uncertainty (e.g. confidence intervals)
☐	☒	For null hypothesis testing, the test statistic (e.g. *F*, *t*, *r*) with confidence intervals, effect sizes, degrees of freedom and *P* value noted<br>*Give P values as exact values whenever suitable.*
☒	☐	For Bayesian analysis, information on the choice of priors and Markov chain Monte Carlo settings
☒	☐	For hierarchical and complex designs, identification of the appropriate level for tests and full reporting of outcomes
☐	☒	Estimates of effect sizes (e.g. Cohen's *d*, Pearson's *r*), indicating how they were calculated

Our web collection on statistics for biologists contains articles on many of the points above.

Software and code

Policy information about availability of computer code

Data collection	In Study 1, participants filled in the questionnaire on the Questionnaire Star platform (https://www.wjx.cn/) through their mobile phones. In Study 2 and Study 3, all stimuli were presented and data was collected using PsychToolBox 3.0.14 (www.psychtoolbox.org) in Matlab 2016a (Mathworks, Natick, MA, USA). FMRI Images in Study 3 were acquired using a 3T Prisma Siemens scanner (Siemens AG, Erlangen, Germany) with a 64-channel head coil at Peking University (Beijing, China). T2-weighted echoplanar images (EPI) were obtained with blood oxygenation level-dependent (BOLD) contrast.
Data analysis	Behavioral data analyses in Study 1 were carried out in IPython/Jupyter Notebook (Python 3.6.8). We used Wordcloud (version 1.8.0, https://amueller.github.io/word_cloud/index.html) and Jieba (version 0.42, https://github.com/fxsjy/jieba) packages to conduct text segmentation, and used collapsed Gibbs sampling implemented in lda package (version 1.1.0, https://lda.readthedocs.io/en/latest/) to conduct Latent Dirichlet Allocation (LDA) based topic modeling on the emotional words of indebtedness.<br><br>Behavioral data analyses in Studies 2 and 3 were carried out in RStudio version 1.1.383. Linear mixed models (LMMs) were conducted using lmeTest package (version 3.1-0). In Study 2, psych package (version 1.9.12), nFactors package (version 2.4.1), and GPArotation package (2014.11-1) were used for the exploratory factor analysis (EFA). In Study 3, confirmatory factor analysis (CFA) was conducted using lavaan package. In both Studies 2 and 3, computational modeling was carried out in Matlab 2016b (https://MathWorks.com) using CosanlabToolbox (https://github.com/ljchang/CosanlabToolbox).<br><br>In Study 3, fMRI data preprocessing and univariate analyses were conducted using Statistical Parametric Mapping software SPM12 (Wellcome Trust Department of Cognitive Neurology, London). Meta-analytic decoding was conducted using the Neurosynth Image Decoder (http://neurosynth.org). FMRI multivariate analyses were performed with our open source Python NLTools package version 0.3.14 (https://nltools.org/).

All the results were plotted using matplotlib, and seaborn 0.9.0 (https://seaborn.pydata.org/index.html). The codes used in the current study are available on github (https://github.com/xiaoxuepsy/Indebtedness).

For manuscripts utilizing custom algorithms or software that are central to the research but not yet described in published literature, software must be made available to editors and reviewers. We strongly encourage code deposition in a community repository (e.g. GitHub). See the Nature Portfolio guidelines for submitting code & software for further information.

Data

Policy information about availability of data

All manuscripts must include a data availability statement. This statement should provide the following information, where applicable:

- Accession codes, unique identifiers, or web links for publicly available datasets
- A description of any restrictions on data availability
- For clinical datasets or third party data, please ensure that the statement adheres to our policy

Data underlying the findings of this study are available on Github (https://github.com/xiaoxuepsy/Indebtedness) and Open Science Framework (https://osf.io/k8rxh/). Meta-analytic decoding was conducted using the meta-analytical images generated by the Neurosynth database (Ref. 61, http://neurosynth.org).

Research involving human participants, their data, or biological material

Policy information about studies with human participants or human data. See also policy information about sex, gender (identity/presentation), and sexual orientation and race, ethnicity and racism.

Reporting on sex and gender	According to self-reported gender, in total, the data of 1,619 (812 females, 18.9 ± 2.0 (SD) years, from Zhengzhou University, China), 51 (33 females, 19.9 ± 1.6 years, from Zhengzhou University, China), 57 (45 females, 20.1 ± 1.8 years, from Zhengzhou University, China), and 53 (29 females, 20.9 ± 2.3 years, from universities at Beijing, China) healthy graduate and undergraduate Chinese Han students were included for Study 1 (experience sampling), Studies 2a and 2b (behavioral studies) and Study 3 (fMRI study), respectively. In addition, 80 participants (45 females, 22.6 ± 2.58 years, from universities at Beijing, China) were recruited for the word classification task to extract emotion-related words in the definition of indebtedness. All the participants were tested only once (i.e., for one experiment). None of the participants reported any history of psychiatric, neurological, or cognitive disorders. Given that the effects of sex and gender were beyond the scope of the current study, the experiments were not specially designed to address related questions, and thus data on sex were not collected, and the gender/sex-related analyses were not conducted in the current article. Informed written consent was obtained from each participant before each experiment.
Reporting on race, ethnicity, or other socially relevant groupings	No race, ethnicity, or other socially relevant groupings was involved.
Population characteristics	See above.
Recruitment	Participants were recruited randomly by paper advertising within Zhengzhou University and universities in Beijing, China. No self-selection bias was involved in the participant recruitment. Although we directly and conceptually replicate our key findings across multiple samples, all of our experiments recruit experimental samples from a Chinese population. It is possible that there are cultural differences in the experience of indebtedness, which may not generalize to other parts of the world. Corresponding discussions have been included in the Discussion section.
Ethics oversight	All of the experiments were carried out in accordance with the Declaration of Helsinki and were approved by the Ethics Committee of the School of Psychological and Cognitive Sciences, Peking University. Informed written consent was obtained from each participant before each experiment.

Note that full information on the approval of the study protocol must also be provided in the manuscript.

Field-specific reporting

Please select the one below that is the best fit for your research. If you are not sure, read the appropriate sections before making your selection.

☐ Life sciences ☒ Behavioural & social sciences ☐ Ecological, evolutionary & environmental sciences

For a reference copy of the document with all sections, see nature.com/documents/nr-reporting-summary-flat.pdf

Behavioural & social sciences study design

All studies must disclose on these points even when the disclosure is negative.

Study description	In this article, we explore these hidden costs by developing and validating a conceptual model of indebtedness across three studies that combine a large-scale questionnaire, an interpersonal game, computational modeling, and neuroimaging. In Study 1 (N = 1619), we explore lay intuitions of indebtedness to test the hypothesis that indebtedness is a mixed feeling comprised of both guilt and obligation, in which participants describe memories of past emotional experiences in a large-scale online questionnaire, using regression analysis and topic modeling. Both qualitative and quantitative methods were used to obtain data in Study 1.

In Study 2 (Study 2a, N = 51; Study 2b, N = 57), we move beyond self-report and focus specifically on how the guilt and obligation components of indebtedness arise and influence behaviors in the context of an interpersonal game. Quantitative data was obtained. In this study, participants receive electrical shocks and anonymous benefactors (co-players) can choose to provide aid to the participants by spending money to reduce the duration of their pain experience. The participants, in turn, have the opportunity to accept or reject this help and also to reciprocate the benefactor's help by sharing some of their own money back. We experimentally manipulate the participants' beliefs about the benefactors' intentions by providing information about whether or not the co-players are aware that the participants have the opportunity to repay after receiving help. We test the hypothesis that appraisals of altruistic intentions produce guilt as well as gratitude (i.e., communal concern) while appraisals of strategic intentions lead to obligation. Building on previous models of other-regarding preferences, we develop computational models to predict reciprocity and help-acceptance decisions respectively in this interpersonal task by quantifying the tradeoff between the latent motivations of self-interest, communal concern (consisting of guilt & gratitude), and obligation based on appraisals induced by the task (Eq. 1).

In Study 3 (N = 53), we provide further validations of the conceptual model by examining the brain processes associated with the two components of indebtedness by scanning an additional cohort of participants playing the interpersonal game while undergoing functional magnetic resonance imaging (fMRI). Quantitative data was obtained. Finally, we construct a neural utility model of indebtedness by applying our computational model directly to multivariate brain patterns to demonstrate that neural signals reflect the tradeoff between these feelings and can be used to predict participants' trial-to-trial reciprocity behavior.

Research sample

According to self-reported gender, in total, the data of 1,619 (812 females, 18.9 ± 2.0 (SD) years, from Zhengzhou University, China), 51 (33 females, 19.9 ± 1.6 years, from Zhengzhou University, China), 57 (45 females, 20.1 ± 1.8 years, from Zhengzhou University, China), and 53 (29 females, 20.9 ± 2.3 years, from universities at Beijing, China) healthy graduate and undergraduate Chinese Han students were included for Study 1 (experience sampling), Studies 2a and 2b (behavioral studies) and Study 3 (fMRI study), respectively. In addition, 80 participants (45 females, 22.6 ± 2.58 years, from universities at Beijing, China) were recruited for the word classification task to extract emotion-related words in the definition of indebtedness. All the participants were tested only once (i.e., for one experiment). None of the participants reported any history of psychiatric, neurological, or cognitive disorders. Given that the effects of sex and gender were beyond the scope of the current study, the experiments were not specially designed to address related questions, and thus data on sex were not collected, and the gender/sex-related analyses were not conducted in the current article. All of the experiments were carried out in accordance with the Declaration of Helsinki and were approved by the Ethics Committee of the School of Psychological and Cognitive Sciences, Peking University. Informed written consent was obtained from each participant before each experiment.

Participants were recruited randomly by paper advertising within Zhengzhou University and universities in Beijing, China. Although we directly and conceptually replicate our key findings across multiple samples, all of our experiments recruit experimental samples from a Chinese population. It is possible that there are cultural differences in the experience of indebtedness, which may not generalize to other parts of the world. Corresponding discussions have been included in the Discussion section.

Meta-analytic decoding was conducted using the meta-analytical images generated by the Neurosynth database (Ref. 61, http://neurosynth.org).

Sampling strategy

Participants were recruited randomly by paper advertising. Although No statistical method was used to predetermine sample size, two lines of evidence suggest that the sample sizes in our experiments are adequate to measure their effect sizes: (1) the key effects in all our experiments achieved acceptable statistical power (larger than 0.8), and (2) our results are repeatable across experiments and by using diverse approaches. First, for the regression analysis suggesting both guilt and obligation ratings contributing to indebtedness rating in Study 1, the estimated statistical power was 0.99. This result was replicated in Studies 2 and 3 (Table S1) and was supported by results of choice question regrading the sources of indebtedness in participants' daily lives and results of topic modeling on the definition of indebtedness in Study 1. Second, as we focused on the two components of indebtedness, guilt and obligation, in Study 2, the values of statistical power for the key effects (i.e., the effect of information regarding benefactor's intention) on guilt and obligation ratings were estimated in Studies 2a and 2b, Study 2a: power-guilt = 0.98, power-obligation = 0.99, Study 2b: power-guilt = 0.97, power-obligation = 0.98. Moreover, we built computational model that was able to successfully capture the participants' reciprocal behaviors after receiving help. The values of statistical power for the regressions using computational model-predicted reciprocity as predictor and observed reciprocity as dependent variable were estimated, Study 2a: power = 0.99, Study 2b: power = 1.00. These results of statistical power analysis indicate that the current sample sizes were adequate to measure the effects of our main manipulation. Importantly, all of the behavioral results in Study 2 were replicated across two behavioral experiments (Studies 2a and 2b) and one fMRI study (Tables S3 and S4), demonstrating the robustness of our results. Third, our computational modeling was supported by evidence at neural level, in which we develop a neural utility model of indebtedness using multivariate patterns of brain activity that captures the tradeoff between these feelings and reliably predicts reciprocity behavior.

Data collection

In Study 1, participants filled in a questionnaire on the Questionnaire Star platform (https://www.wjx.cn/) through their mobile phones with no communication with any researcher.

In Study 2 and Study 3, all stimuli were presented and data was collected using PsychToolBox 3.0.14 (www.psychtoolbox.org) in Matlab 2016a (Mathworks, Natick, MA, USA) on computers. We applied within-subject designs and thus did not involve the issues of participant grouping, randomization or blinding procedure. The trial order for each participant was randomized.

In Study 2a and Study 2b, five to seven participants came to the experiment room together. Participants sat in front of their computers, were separated by partitions, and completed the task independently during the experiment. A researcher sat two meter away waiting for the participants to complete the experiment. There were no other people present during the experiment.

In Study 3, participants lie down in the MRI scanner to complete the task independently. A researcher and a scanning staff observed the participants' real-time head movements and behaviors outside the scanning room. No one else was present inside or outside the scanning room.

Timing

Study 1: September 3rd to September 30th, 2017.

	Study 2: September 3rd to 10th, 2017.<br>Study 3: February 28th to June 3rd, 2018.
Data exclusions	For Study 1 (large-scale sampling), 1,808 graduate and undergraduate students were recruited. One hundred and eighty-nine participants were excluded from data analysis because of filling in irrelative information in the essay question, leaving 1,619 participants for data analysis. For Study 2a (behavioral study), 7 participants were excluded due to equipment malfunction. For Study 2b (behavioral study), 3 participants were excluded due to failing to respond in more than 10 trials. For Study 3 (fMRI study), 4 participants with excessive head movements (>2mm) were excluded.
Non-participation	No participants dropped out/declined participation.
Randomization	We applied within-subject design. All participant went through all conditions and were not allocated into any experimental group. The order of conditions were fully randomized.

Reporting for specific materials, systems and methods

We require information from authors about some types of materials, experimental systems and methods used in many studies. Here, indicate whether each material, system or method listed is relevant to your study. If you are not sure if a list item applies to your research, read the appropriate section before selecting a response.

Materials & experimental systems

n/a	Involved in the study
[x]	[ ] Antibodies
[x]	[ ] Eukaryotic cell lines
[x]	[ ] Palaeontology and archaeology
[x]	[ ] Animals and other organisms
[x]	[ ] Clinical data
[x]	[ ] Dual use research of concern
[x]	[ ] Plants

Methods

n/a	Involved in the study
[x]	[ ] ChIP-seq
[x]	[ ] Flow cytometry
[ ]	[x] MRI-based neuroimaging

Magnetic resonance imaging

Experimental design

Design type	This is an event-related task-based fMRI study.
Design specifications	There are 3 blocks, each contain 18 trials. One trial ranges from 29 to 45 seconds depending on each participant's response times. Inter-trial interval varies from 2 to 6 sec, with a mean value of 4 sec.
Behavioral performance measures	During the scanning, behavioral performances are the participants' evaluations on how much they thought the co-player expected him/her to reciprocate and the money that the participants allocated to the co-player. Post-scan behavioral performances include participants' ratings on how much they believed the benefactor cared for them, as well as their feelings of gratitude, indebtedness, sense of obligation, and guilt when they received the help for each trial.

Acquisition

Imaging type(s)	Functional MRI
Field strength	3T
Sequence & imaging parameters	Images were acquired using a 3T Prisma Siemens scanner (Siemens AG, Erlangen, Germany) with a 64-channel head coil at Peking University (Beijing, China). T2-weighted echoplanar images (EPI) were obtained with blood oxygenation level-dependent (BOLD) contrast. Sixty-two transverse slices of 2.3 mm thickness that covered the whole brain were acquired using multiband EPI sequence in an interleaved order (repetition time = 2000 ms, echo time = 30 ms, field of view = 224×224 mm2, flip angle = 90°).
Area of acquisition	Whole brain

Diffusion MRI [ ] Used [x] Not used

Preprocessing

Preprocessing software	The fMRI data preprocessing was conducted using Statistical Parametric Mapping software SPM12 (Wellcome Trust Department of Cognitive Neurology, London). Images were slice-time corrected, motion corrected, resampled to 3 mm × 3 mm × 3 mm isotropic voxels, and normalized to MNI space using the EPInorm approach in which functional images are aligned to an EPI template, which is then nonlinearly warped to stereotactic space (see Ref. 121). Images were then spatially smoothed with an 8 mm FWHM Gaussian filter, and temporally filtered using a high-pass filter with a cutoff frequency of

	1/128 Hz.
Normalization	Images were slice-time corrected, motion corrected, resampled to 3 mm × 3 mm × 3 mm isotropic voxels, and normalized to MNI space using the EPInorm approach in which functional images are aligned to an EPI template, which is then nonlinearly warped to stereotactic space (see Ref. 121).
Normalization template	EPI template
Noise and artifact removal	Six rigid-body head movement parameters were included in GLMs as regressors of no interest.
Volume censoring	Four participants with excessive head movements (>2mm) were excluded. Six rigid-body head movement parameters were included in GLMs as regressors of no interest to control for head movements. No volume censoring process was conducted.

Statistical modeling & inference

Model type and settings

First, for univariate fMRI Analyses, we used a model-based fMRI analytic approach (Ref. 59) to identify brain regions that parametrically tracked different components of the computational model for reciprocity during the Outcome period of the task (5s; Fig. 3b), where participants learned about the benefactor's decision to help. To ensure that each hypothesis tested had maximum variance, we chose to separately test each hypothesis using a separate model to minimize issues with multicollinearity. GLM 1 identified reciprocity related brain responses based on the parametric modulator of participant's reciprocity behavior DB. GLM 2 identified brain responses related to communal concern based on the parametric modulator of the participant's appraisal of perceived care ωB. GLM 3 identified brain responses related to obligation, which we modeled as a linear contrast of the participant's second-order belief of the benefactor's expectation for repayment EB''. We chose to use the appraisals rather than the UCommunal and the UObligation terms, as those terms create costs based on the squared deviation from reciprocity behavior, which results in a large proportion of trials where the deviations are near zero as a result of participant's decisions, making them inefficient for parametric analysis to capture how successfully participants behaved in accordance with their feelings. Instead, ωB and EB'' better captured the inferences that comprised participants' feelings and were more suitable for testing our hypotheses about brain responses.

Regressors for GLM1 and GLM 2 included: (a) Outcome period (onset of the presentation of the benefactor's decision, 5s) with the corresponding parametric modulator, (b) Information period (onset of the presentation of the benefactor's picture and extra information regarding intention, 4s), (c) Second-order belief rating period (starting from the time the rating screen presented and spanning to the time that the participant made choice), (d) Allocation period (starting from the time the rating screen presented and spanning to the time that the participant made choice), (e) Missed responses (the missing decision period for second-order belief or allocation, 8s), and (f) six head motion realignment parameters. Contrasts were defined as the positive effect of the parametric modulator of interest.

For GLM3, because our computational model's representation of second order beliefs EB" had a non-normal distribution (zero in Repayment impossible condition and linear increase in Repayment possible condition, Eq. 3), we constructed a piecewise linear contrast, instead of linear parametric analysis. This entailed creating four separate regressors modeling different parts of the function during the Outcome period: (1) Repayment impossible, (2) Repayment possible and low benefactor's cost (i.e., 4, 6, or 8), (3) Repayment possible and medium benefactor's cost (i.e., 10, 12, or 14), (4) Repayment possible and high benefactor's cost (i.e., 16, 18, or 20). Subsequently, for each participant, we constructed a contrast vector of c = [-6, 1, 2, 3]. This piecewise linear contrast ensures that brain responses to the Repayment impossible trials are lower than all of the Repayment possible trials. We have successfully used this approach in previous work modeling guilt using similar Psychological Game Theoretic utility models (Ref. 54).

For all GLMs, events in each regressor were convolved with a double gamma canonical hemodynamic response function. Second-level models were constructed as one-sample t tests using contrast images from the first-level models. For whole brain analyses, all results were corrected for multiple comparisons using cluster correction $p < 0.05$ with a cluster-forming threshold of $p < 0.001$, which attempts to control for family wise error (FWE) using Gaussian Random Field Theory (Ref, 122).

Second, we constructed a neural utility model by combining our computational model for reciprocity with multivariate pattern analysis (MVPA, Ref. 125). First, using principal components regression with 5-fold cross-validation, we trained two separate multivariate whole-brain models predictive of communal concern (ωB) and obligation (EB'') terms in our behavioral model separately for each participant (Ref. 71-73). This entailed first performing temporal data reduction by estimating single-trial beta maps of the Outcome period for each participant. Then for each participant, we separately predicted ωB and EB'' from a vectorized representation of the single trial beta maps. Because these models have considerably more voxel features (~328k) then trial observations, we performed a principal components analysis to reduce the feature space and used the principal components to predict the model appraisal representations (e.g., ωB and EB''). We then back-projected the estimated beta components from the regression back into the full voxel feature space, and then back to 3-D space. We have previously demonstrated that this approach is effective in reliably mapping the independent contribution of each voxel in the brain to a psychological state to identify the neural representations of affective states (Ref. 72,126,127). For each whole-brain model, we extracted the cross-validated prediction accuracy (r value) for each participant, conducted r-to-z transformation, and then conducted a one-sample permutation t-test to evaluate whether each model was able to significantly predict the corresponding term.

We used the cross-validated models to generate predictions for each trial for each participant and then used brain-predicted communal concern (ωB) and obligation (EB") feelings as inputs to our computational model of reciprocity behavior (Model 1.1, Eq. 9) instead of the original terms. We estimated the θ values (i.e., weight on greed) and ϕ weighting parameters (i.e., relative trade-off between on communal concern and obligation) using the same procedure described in the behavioral computational modeling section by fitting the neural utility model directly to participant's reciprocity behavior by minimizing the SSE (Eq. 10).

Effect(s) tested

In univariate analysis, second-level models were constructed as one-sample t tests using contrast images from the first-level models to identify brain regions that tracked each component in the computational model: the amount of reciprocity, communal concern (depended on ωB) and obligation (depended on EB'').

In multivariate analysis, for each whole-brain model, we extracted the cross-validated prediction accuracy (r value) for each participant, conducted r-to-z transformation, and then conducted a one-sample permutation t-test to evaluate whether each model was able to significantly predict the corresponding term.

We used the cross-validated models to generate predictions for each trial for each participant and then used brain-predicted communal concern (ωB) and obligation (EB") feelings as inputs to our computational model of reciprocity behavior (Model 1.1, Eq. 9) instead of the original terms. We estimated the θ values (i.e., weight on greed) and ϕ weighting parameters (i.e., relative trade-off between on communal concern and obligation) using the same procedure described in the behavioral computational modeling section by fitting the neural utility model directly to participant's reciprocity behavior by minimizing the SSE (Eq. 10).

As a benchmark for our neural utility model, we were interested in determining how well we could predict participant's reciprocity behavior directly from brain activity. We used the same training procedure described above, but predicted trial-to-trial reciprocity behavior using principal components regression separately for each participant. In theory, this should provide a theoretical upper bound of the best we should be able to predict reciprocity behavior using brain activity. If our neural utility model is close, then it means that we are able to predict reciprocity behavior using brain representations of communal concern and obligation as well as the optimal linear weighting of brain weights that can predict trial-to-trial reciprocity behavior. To determine the importance of the participant-specific model parameters, we ran a permutation test to determine how well we could predict reciprocity behavior for each participant using parameters from a randomly selected different participant. We ran 5,000 permutations to generate a null distribution of average prediction accuracy after randomly shuffling the participant weights. The empirical p-value is the proportion of permutations that exceed our average observed correlation.

Specify type of analysis: ☒ Whole brain ☐ ROI-based ☐ Both

Statistic type for inference

(See Eklund et al. 2016)

For whole brain analyses, all results were corrected for multiple comparisons using cluster correction $p < 0.05$ with a cluster-forming threshold of $p < 0.001$, which attempts to control for family wise error (FWE) using Gaussian Random Field Theory. This approach attempts to estimate the number of independent spatial resels or resolution elements in the data necessary to control for FWE. This calculation requires defining an initial threshold to determine the Euler Characteristic of the data. It has been demonstrated that an initial threshold of $p < 0.001$ does a reasonable job of controlling for false positives at 5% using this approach (Ref. 122).

Correction

For whole brain analyses, all results were corrected for multiple comparisons using cluster correction $p < 0.05$ with a cluster-forming threshold of $p < 0.001$, which attempts to control for family wise error (FWE) using Gaussian Random Field Theory. This approach attempts to estimate the number of independent spatial resels or resolution elements in the data necessary to control for FWE. This calculation requires defining an initial threshold to determine the Euler Characteristic of the data. It has been demonstrated that an initial threshold of $p < 0.001$ does a reasonable job of controlling for false positives at 5% using this approach (Ref. 122).

Models & analysis

n/a	Involved in the study
☒	☐ Functional and/or effective connectivity
☒	☐ Graph analysis
☐	☒ Multivariate modeling or predictive analysis

Multivariate modeling and predictive analysis

We constructed a neural utility model by combining our computational model for reciprocity with multivariate pattern analysis (MVPA, Ref. 125). First, using principal components regression with 5-fold cross-validation, we trained two separate multivariate whole-brain models predictive of communal concern (ωB) and obligation (EB'') terms in our behavioral model separately for each participant (Ref. 71-73). This entailed first performing temporal data reduction by estimating single-trial beta maps of the Outcome period for each participant. Then for each participant, we separately predicted ωB and EB'' from a vectorized representation of the single trial beta maps. Because these models have considerably more voxel features (~328k) then trial observations, we performed a principal components analysis to reduce the feature space and used the principal components to predict the model appraisal representations (e.g., ωB and EB''). We then back-projected the estimated beta components from the regression back into the full voxel feature space, and then back to 3-D space. We have previously demonstrated that this approach is effective in reliably mapping the independent contribution of each voxel in the brain to a psychological state to identify the neural representations of affective states (Ref. 72,126,127). For each whole-brain model, we extracted the cross-validated prediction accuracy (r value) for each participant, conducted r-to-z transformation, and then conducted a one-sample permutation t-test to evaluate whether each model was able to significantly predict the corresponding term.

We used the cross-validated models to generate predictions for each trial for each participant and then used brain-predicted communal concern (ωB) and obligation (EB") feelings as inputs to our computational model of reciprocity behavior (Model 1.1, Eq. 9) instead of the original terms. We estimated the θ values (i.e., weight on greed) and ϕ weighting parameters (i.e., relative trade-off between on communal concern and obligation) using the same procedure described in the behavioral computational modeling section by fitting

the neural utility model directly to participant's reciprocity behavior by minimizing the SSE (Eq. 10).

As a benchmark for our neural utility model, we were interested in determining how well we could predict participant's reciprocity behavior directly from brain activity. We used the same training procedure described above, but predicted trial-to-trial reciprocity behavior using principal components regression separately for each participant. In theory, this should provide a theoretical upper bound of the best we should be able to predict reciprocity behavior using brain activity. If our neural utility model is close, then it means that we are able to predict reciprocity behavior using brain representations of communal concern and obligation as well as the optimal linear weighting of brain weights that can predict trial-to-trial reciprocity behavior. To determine the importance of the participant-specific model parameters, we ran a permutation test to determine how well we could predict reciprocity behavior for each participant using parameters from a randomly selected different participant. We ran 5,000 permutations to generate a null distribution of average prediction accuracy after randomly shuffling the participant weights. The empirical p-value is the proportion of permutations that exceed our average observed correlation.

Finally, we were interested in evaluating how well we could estimate how much each participant had a relative preference for communal concern or obligation by computing the relative spatial alignment of their communal and obligation predictive spatial maps with their reciprocity predictive spatial map.
